# Perinatal health effects of herbicides exposures in the United States: the Heartland Study, a Midwestern birth cohort study

**DOI:** 10.1186/s12889-023-17171-9

**Published:** 2023-11-22

**Authors:** Marlaina Freisthler, Paul W. Winchester, Heather A. Young, David M. Haas

**Affiliations:** 1 Department of Environmental and Occupational Health, Milken Institute of Public Health, George Washington University, 950 New Hampshire Ave NW #2, Washington, DC 20052 USA; 2 Neonatal-Perinatal Medicine, Riley Children’s Hospital, Indiana University School of Medicine, 699 Riley Hospital Dr RR 208, Indianapolis, IN 46202 USA; 3 Franciscan Health, Indianapolis, 8111 South Emerson Avenue, Indianapolis, IN 46237 USA; 4 Department of Epidemiology, Milken Institute for Public Health, George Washington University, 950 New Hampshire Ave NW #2, Washington, DC 20052 USA; 5 Department of Obstetrics and Gynecology, Indiana University School of Medicine, 550 N. University Blvd, Indianapolis, IN UH2440 USA

**Keywords:** Herbicides, Glyphosate, Birth cohort, Reproductive impacts

## Abstract

**Background:**

The objective of the Heartland Study is to address major knowledge gaps concerning the health effects of herbicides on maternal and infant health. To achieve this goal, a two-phased, prospective longitudinal cohort study is being conducted. Phase 1 is designed to evaluate associations between biomarkers of herbicide concentration and pregnancy/childbirth outcomes. Phase 2 is designed to evaluate potential associations between herbicide biomarkers and early childhood neurological development.

**Methods:**

People (target enrollment of 2,000) who are seeking prenatal care, are ages 18 or older, and are ≤ 20 + 6 weeks gestation will be eligible for recruitment. The Heartland Study will utilize a combination of questionnaire data and biospecimen collections to meet the study objectives. One prenatal urine and buccal sample will be collected per trimester to assess the impact of herbicide concentration levels on pregnancy outcomes. Infant buccal specimens will be collected post-delivery. All questionnaires will be collected by trained study staff and clinic staff will remain blinded to all individual level research data. All data will be stored in a secure REDCap database.

Hospitals in the agriculturally intensive states in the Midwestern region will be recruited as study sites. Currently participating clinical sites include Indiana University School of Medicine- affiliated Hospitals in Indianapolis, Indiana; Franciscan Health Center in Indianapolis, Indiana; Gundersen Lutheran Medical Center in La Crosse, Wisconsin, and University of Iowa in Iowa City, Iowa. An anticipated 30% of the total enrollment will be recruited from rural areas to evaluate herbicide concentrations among those pregnant people residing in the rural Midwest.

Perinatal outcomes (e.g. birth outcomes, preterm birth, preeclampsia, etc.) will be extracted by trained study teams and analyzed for their relationship to herbicide concentration levels using appropriate multivariable models.

**Discussion:**

Though decades of study have shown that environmental chemicals may have important impacts on the health of parents and infants, there is a paucity of prospective longitudinal data on reproductive impacts of herbicides. The recent, rapid increases in herbicide use across agricultural regions of the United States necessitate further research into the human health effects of these chemicals, particularly in pregnant people. The Heartland Study provides an invaluable opportunity to evaluate health impacts of herbicides during pregnancy and beyond.

**Trial registration:**

The study is registered at clinicaltrials.gov, NCT05492708 with initial registration and release 05 August, 2022.

**Supplementary Information:**

The online version contains supplementary material available at 10.1186/s12889-023-17171-9.

## Background

The prevalence of adverse maternal and neonatal outcomes has been increasing in the US, and the causes of these increases are incompletely understood. For example, the incidence of pre-eclampsia has been increasing in the United States over the past twenty years, and currently affects between 3 and 6 percent of all births [1]. Pre-eclampsia is a well-known risk factor for preterm birth [2] and other adverse maternal and infant outcomes, including longer-term cardiovascular disease [3, 4]. Approximately one in ten infants born in the US is born preterm (< 37 weeks gestation) [5] and after a previous period of decline, rates of preterm birth and low birthweight have been on the rise [6]. Gestational diabetes, a condition associated with both neonatal morbidity and long-term metabolic implications for women [7], is also increasing in incidence and is estimated to affect at least 1 in 20 births [8]. Birth defects, the leading cause of neonatal mortality, affect an estimated 3–6% of all births, with rates of defects on the rise [9]. Taken together, these conditions pose considerable burdens for both maternal and neonatal health [10].

While these concerning trends have been observed in maternal and neonatal health, evidence of the contribution of environmental exposures to adverse health outcomes has been mounting [11–14], with pesticides implicated as an exposure of particular concern [15–19].

Herbicides are a class of pesticides used to control weeds. In order to control the problem of herbicide resistance, next generation weed control methods have been developed that employ genetically modified seeds that are tolerant to repeated applications of multiple herbicides [20]. In addition to relying on multiple herbicides to achieve weed control, next generation methods depend on herbicide applications occurring with greater frequency and over longer time periods than in traditional weed control regimens [21]. As a result, annual applications of glyphosate and glufosinate, as well as atrazine, dicamba, and 2,4-dichlorophenoxyacetic acid (2,4-D) (herbicides commonly coupled with glyphosate/glufosinate) are increasing, particularly in areas of the US that are home to intensive agricultural activity, such as the Midwest and Southeast [22]. As genetically modified weed control methods become more widely utilized, human exposures to herbicides are likely to increase [23]. There is an association between residence near agricultural land and increased concentrations of pesticides [24]. Rurality, a term that incorporates the collective, compositional, and contextual differences associated with living outside an urban area [25], is a known contributor to infant mortality risk [26, 27].

### Prior studies evaluating herbicides and maternal and child health

Herbicides have long been implicated in adverse reproductive outcomes [28–32]. A variety of well-known U.S. birth cohort studies have considered the reproductive impact of various pesticide exposures [33–36], but these studies have primarily focused on persistent insecticides. With respect to herbicides, while longitudinal US birth cohorts exist evaluating various proxy measures of exposure (e.g., self-report, geographical proximity to application, and occupational exposure estimates) [37], few US birth cohorts have considered the reproductive impact of herbicides using longitudinal biomonitoring. Table 1 lists epidemiologic studies that have reported on associations between herbicide exposure and reproductive outcomes. Several recent studies utilizing advances in biomonitoring for exposure assessment have demonstrated associations between herbicide concentrations and adverse birth outcomes [38–40]. Other associations between herbicides and adverse reproductive outcomes observed in animal models have not been adequately assessed in human biomonitoring studies, perhaps in part due to a lack of biomonitoring capability [41] and examples of these outcomes are listed in Table 2. As far as we are aware, no longitudinal birth cohort studies evaluating associations between multiple herbicide concentration biomarkers and reproductive and developmental impacts have been completed in the US despite widespread and increasing herbicide use [21] and nearly ubiquitous environmental exposure to herbicides [39, 42].

**Table 1:** Key human studies informing Heartland Study hypotheses

Study	Authors/Year	Herbicide	n	Exposure Assessment	Outcome Associations	Effect Size
Urinary glyphosate concentration in pregnant women in relation to length of gestation	(Lesseur et al., 2022)	Glyphosate and AMPA	163	Biomarkers in Second trimester urine samples	Shortened length of gestation in spontaneous births	Glyphosate: HR = 1.31, (95%CI 1.00–1.71) AMPA: HR = 1.32 (95%CI: 1.00–1.73)
Prenatal exposure to organophosphate and pyrethroid insecticides and the herbicide 2,4-dichlorophenoxyacetic acid and size at birth in urban pregnant women	(Balalian et al., 2021)	2,4-D	270	Urinary biomarkers of 2,4-D	Inverse association between concentration and head circumference	2,4-D was associated with smaller head circumference in the second (β = − 1.57; (95%CI: − 2.74, − 0.39) and third (β = − 1.74, (95%CI: − 2.98, − 0.49) categories of concentration in comparison to the first category
Prenatal exposure to pesticides and risk of preeclampsia among pregnant women: Results from the ELFE cohort	(Enderle et al., 2021)	Herbicides	17,376	Self-reported home application	Pre-eclampsia	Women receiving antihypertensive treatment for pre-eclampsia compared to women without pre-eclampsia (aOR = 2.20, (95%CI: 1.23, 3.93)
Maternal urinary levels of glyphosate during pregnancy and anogenital distance in newborns in a US multicenter pregnancy cohort	(Lesseur et al., 2021)	Glyphosate and AMPA	94	Second trimester urine biomarker	Anogenital distance in female offspring	After adjusting for confounders, increased AMPA was associated with longer AGD in female infants, β = 1.96, (95%CI 0.44, 3.5)
Prenatal Exposure to Glyphosate and Its Environmental Degradate, Aminomethylphosphonic Acid (AMPA), and Preterm Birth: A Nested Case–Control Study in the PROTECT Cohort (Puerto Rico)	(Silver et al., 2021)	Glyphosate and AMPA	247	Multiple second trimester urine biomarkers	Increased odds of preterm birth	aORs for an interquartile range increase in exposure at Visit 3 were 1.35 (95% CI: 0.99, 1.83) and 1.67 (95% CI: 1.26, 2.20) for GLY and AMPA, respectively
Prenatal and infant exposure to ambient pesticides and autism spectrum disorder in children: population based case–control study	(Ehrenstein et al., 2019)	Glyphosate	41,292	GIS, California Pesticide Use records	Autism spectrum disorder	ASD: Glyphosate OR = 1.16, (95% CI 1.06, 1.27); ASD with intellectual disability: Glyphosate OR = 1.33 (95%CI 1.05,1.69)
Atrazine Contamination of Drinking Water and Adverse Birth Outcomes in Community Water Systems with Elevated Atrazine in Ohio, 2006–2008	(Almberg et al., 2018)	Atrazine	14,445	Mean gestational and trimester-specific atrazine concentrations were calculated from EPA water quality monitoring data	Term low birth weight	aOR 1.27 (95% CI 1.10, 1.45)
Glyphosate exposure in pregnancy and shortened gestational length: a prospective Indiana birth cohort study	(Parvez et al., 2018)	Glyphosate	71	Urine biomarkers of exposure	Shortened length of gestation	GLY urine levels were significantly correlated with shortened gestational lengths (r = − 0.28, p = 0.02)
Exposure to pistachio pesticides and stillbirth: a case–control study	(Razi et al., 2016)	Proximity to pistachio orchards as a proxy for organo-phosphate pesticide exposure	375	Self-report	Stillbirth	In mothers living in pistachio gardens the OR = 14.1 (95% CI, 3.3, 63.4) In mothers exposed to sprayed pesticides, the OR = 5.0 (95% CI, 1.2, 28.6)
County-level pesticide use and risk of shortened gestation and preterm birth	(Winchester et al., 2016)	Pesticides	7,940,794	California Pesticide Use records	Preterm birth, low birthweight percentile	Counties with higher pesticide use were associated with higher PTB (low 8.59 ± 0.11%, moderate 9.25 ± 0.07%, high 10.0 ± 0.06%, p's < 0.001) and shorter gestations (low 39.197 ± 0.014 weeks, moderate 39.126 ± 0.011 weeks, high 39.049 ± 0.011 weeks, p's < 0.001)
Maternal Residential Atrazine Exposure and Risk for Choanal Atresia and Stenosis in Offspring	(Agopian, Cai, et al., 2013)	Atrazine	4092	USGS estimated residential atrazine exposure	Choanal atresia and stenosis	Mothers with high levels of residential atrazine exposure had an increased risk of choanal atresia or stenosis with an aOR = 1.79, (95% CI 1.17, 2.74)
Maternal Residential Atrazine Exposure and Gastroschisis by Maternal Age	(Agopian, Langlois, et al., 2013)	Atrazine	95,551	USGS estimated residential atrazine exposure	Gastroschisis	Risk for gastroschisis in offspring was significantly increased for women ≥ 25 years with high levels of residential atrazine exposure compared to low aOR = 1.97, (CI: 1.19–3.26)
Atrazine Exposure in Public Drinking Water and Preterm Birth	(Rinsky et al., 2012)	Public drinking water atrazine data	71,768	Public drinking water data	Preterm birth	Increase in the odds of preterm birth noted in those women living in counties with the highest level of atrazine exposure, OR = 1.26, (95% CI 1.19, 1.32)
Drinking-water herbicide exposure in Indiana and prevalence of small-for-gestational-age and preterm delivery	(Ochoa-Acuña et al., 2009)	Drinking water nitrate and atrazine levels	24,154	Indiana geocoding logs	Small for gestational age	Mean atrazine concentrations over the entire pregnancy > 0.644 microg/L were associated with higher SGA prevalence than in the control group, adjusted PR = 1.14, (95% CI 1.03, 1.24)
Pre- and post-conception pesticide exposure and the risk of birth defects in an Ontario farm population	(Weselak et al., 2008)	Self-Reported dicamba use	3,412	Self-report	Birth defects	Pre-conception exposure to dicamba in males increased the risk of birth defects, OR = 2.42, (95% CI 1.06, 5.53)
Pesticide Exposure and Self-Reported Gestational Diabetes Mellitus in the Agricultural Health Study	(Saldana et al., 2007)	Self-reported pesticide use	11,273	Self-report from FFH	Gestational diabetes mellitus	Women who reported agricultural pesticide exposure (mixing or applying pesticides to crops or repairing pesticide application equipment) during pregnancy were more likely to report GDM, OR = 2.2 (95% CI 1.5, 3.3)
Spontaneous Abortion in Spouses of Greenhouse Workers Exposed to Pesticides	(Petrelli et al., 2003)	Occupational exposure to greenhouse atrazine	184	Self-report	Spontaneous abortion	The OR adjusted for age, smoking, and education of both partners and for spouse’s type of work and time between the pregnancy and the interview yielded an OR = 11.8, CI: 2.3–59.6
Birth malformations and other adverse perinatal outcomes in four U.S. wheat-producing states	(Schreinemachers, 2003)	Geographically estimated chlorophenoxy herbicide exposures	43,634	Geographic estimation	Birth malformations	Significant increases in birth malformations were observed for the circulatory/respiratory category for the combined sexes, OR = 1.65, (95% CI 1.07, 2.55)
An exploratory analysis of the effect of pesticide exposure on the risk of spontaneous abortion in an Ontario farm population	(Arbuckle et al., 2001)	Self-reported pesticide use	3,936	Self-report	Late spontaneous abortions	We observed moderate increases in risk of early abortions for preconception exposures to phenoxy acetic acid herbicides (OR) = 1.5, 95% CI, 1.1–2.1

Legend: *CI* Confidence Interval, *HR* Hazard Ratio, *OR* Odds Ratio, *Aor* Adjusted Odds Ratio, *β* Beta Coefficient r = Spearman’s rank-based correlation

**Table 2:** Key animal studies informing Heartland Study hypotheses

Perinatal exposure to a glyphosate-based herbicide impairs female reproductive outcomes and induces second-generation adverse effects in Wistar rats	(Milesi et al., 2021)	Glyphosate	Increased resorptions in rats	Perinatal exposure to low doses of a GBH impaired female reproductive performance and induced fetal growth retardation and structural congenital anomalies in F2 offspring
Epigenetic disruption of estrogen receptor alpha is induced by a glyphosate-based herbicide in the preimplantation uterus of rats	(Rossetti et al., 2021) (Lorenz et al., 2019)	Glyphosate	Epigenetic changes in rodents	Perinatal exposure to a GBH causes long-term epigenetic disruption of the uterine estrogen-receptor α gene, which could be associated with the GBH-induced implantation failures
Maternal glyphosate exposure causes autism-like behaviors in offspring through increased expression of soluble epoxide hydrolase (sEH)	(Pu et al., 2020)	Glyphosate	Autism-like traits in mice	The glyphosate exposures used here exceed any reasonable dietary, environmental, or occupational exposure, but they indicate that increased sEH plays a role in ASD-like behaviors in offspring
Assessment of Glyphosate Induced Epigenetic Transgenerational Inheritance of Pathologies and Sperm Epimutations: Generational Toxicology	(Kubsad et al., 2019)	Glyphosate	Epigentic changes in rats	Support for an association between glyphosate exposure and transgenerational germline epimutations
Reproductive toxicity of Roundup herbicide exposure in male albino rats	(Owagboriaye et al., 2017)	Glyphosate	Decreased sperm count and motility, increased abnormal sperm morphology	The lowest sperm count was observed in rats exposed to Roundup at 248.4 mg/kg bodyweight of glyphosate
Effects of neonatal exposure to a glyphosate-based herbicide on female rat reproduction	(Ingaramo et al., 2016)	Glyphosate	Increased resorption sites in rats	Alterations in endometrial decidualization might be the mechanism of GBH-induced post-implantation embryo loss
Pre- and postnatal exposure to low dose glufosinate ammonium induces autism-like phenotypes in mice	(Laugeray et al., 2014)	Glufosinate	Autism phenotypes in mice	A neurobehavioral test battery revealed significant effects of GLA maternal exposure on early reflex development, pup communication, affiliative behaviors, and preference for social olfactory cues, but emotional reactivity and emotional memory remained unaltered. These behavioral alterations showed a striking resemblance to changes seen in animal models of Autistic Spectrum Disorders

Legend: *CI* Confidence Interval, *HR* Hazard Ratio, *OR* Odds Ratio, *aOR* Adjusted Odds Ratio, *β *Beta Coefficient r = Spearman’s rank-based correlation

### Objective

The objective of the Heartland Study is to address major knowledge gaps concerning the health effects of herbicides on maternal and infant health. To achieve this goal, a prospective longitudinal cohort study is being conducted to evaluate the associations between environmental concentrations of herbicides during and after pregnancy and reproductive health outcomes. The study will measure multiple herbicide concentrations among pregnant Midwesterners and their partners to evaluate associations with pregnancy and childbirth outcomes and child development.

### Design of the heartland study

The Heartland Study is occurring in multiple phases. Phase 1 evaluates associations between herbicide biomarkers of exposure and pregnancy and childbirth outcomes, as well as maternal and infant epigenetic changes. Phase 2 evaluates associations between biomarkers of herbicide concentration during intrauterine development and early childhood developmental outcomes, with a focus on neurological outcomes. This report details the methods and measurements of Phase 1. The study is registered on clinical trials.gov (NCT05492708).

In Phase 1, it is hypothesized that increased use of herbicides as measured from agricultural use data will correspond with herbicide biomarker concentrations collected during pregnancy; that herbicide detections/concentrations will be associated with adverse pregnancy and childbirth health outcomes, including gestational hypertension/pre-eclampsia, gestational diabetes, spontaneous abortion/stillbirth, congenital anomalies, shortened gestation, and low birth weight percentile; and that herbicide concentration will be associated with differences in epigenetic biomarkers.

## Methods

### Recruitment

Hospitals located in one of the 13 Heartland Study region states (Arkansas, Illinois, Indiana, Iowa, Kansas, Michigan, Minnesota, Missouri, Nebraska, North Dakota, Ohio, South Dakota, or Wisconsin) will be recruited as affiliated clinical sites. Currently participating clinical sites include Indiana University School of Medicine- affiliated Hospitals in Indianapolis, Indiana; Franciscan Health Center in Indianapolis, Indiana; and Gundersen Lutheran Medical Center in La Crosse, Wisconsin, and University of Iowa in Iowa City, Iowa. Pregnant persons attending obstetric clinics affiliated with a clinical site hospital will be recruited, although other pregnant peopleliving in Heartland Study states are also eligible to participate.

The target enrollment is 2000 pregnant dyads. To evaluate the distribution of herbicide concentrations among Midwestern pregnancies, a target of 30% of participants will be recruited from rural areas, categorized as those areas outside of urban area centers according to US census designations [43]. Pregnant persons ages 18 or older at time of recruitment who are being seen at prenatal clinics and who are ≤ 20 + 6 weeks gestation will be recruited. Best clinical estimate of gestational age will be utilized for recruitment purposes [44]. Both singleton and twin pregnancies will be included. The other putative biological parent will also be invited to enroll as a secondary participant, subject to the primary participant’s consent. Exclusion criteria include the primary participant not being fluent in and/or able to fully understand, read, write, or speak the English or other approved languages, or inability to provide written informed consent. Trained study personnel will screen for eligibility, explain the study purpose and protocol, and administer informed consent. Approval for the Heartland Study protocol and procedures will be obtained from each clinical site hospital’s local governing Institutional Review Board. If materials are developed in other languages, such as Spanish, persons who are able to fully understand, read, write, or speak those languages will be eligible for enrollment.

### Study protocol

A common protocol and manual of operations have been developed covering all aspects of the study for utilization by all sites. Obstetric and newborn care are delivered according to local practices and standards at the individual clinical sites. Trained study personnel perform all study-related procedures. Training procedures and evaluation methods have been developed and are utilized under the supervision of the Study Principal Investigators, to ensure consistency between sites. Within each site, implementation of general study procedure training is the responsibility of the Site Principal Investigator and Study Coordinator for each site. Quality control checks (via re-abstraction) are performed for primary outcomes by site Principal Investigators on randomly selected charts with and without adverse outcomes.

Study visits typically coincide with regular obstetrical care visits. Attempts are made to collect one prenatal urine sample and one prenatal buccal sample at each pregnancy trimester to assess urinary concentration levels and epigenetic alterations that may be occurring throughout pregnancy. Pregnancy trimesters are defined as first trimester (through 13 weeks), second trimester (14–27 weeks), and third trimester (28–42 weeks). Biospecimen collection and questionnaire data collection are administered separately from obstetric and neonatal clinical care. Biospecimen collection timing was chosen to facilitate evaluation of longitudinal/trimester effect of associations between herbicide concentrations and outcomes. Study adherence is incentivized by compensation to participants for their time and effort. The amount and types of compensation are determined in advance and are consistent across all sites throughout the study.

Several questionnaire instruments are utilized during the study. The Supplementary file contains the questionnaire instruments used during the study. Pre-conception questionnaires are administered to participants to record demographics, reproductive and medical histories, residential and occupational/recreational histories, and environmental exposures. Stress during pregnancy is a known preterm delivery risk factor (Roy-Matten et al., 2011). Additionally, stress is known to compromise the immune system, which may increase vulnerability to epigenetic alterations induced by pesticide exposures (Palma-Gudiel et al., 2015). The maternal perceived stress scale (PSS) is administered throughout pregnancy to assess pregnancy stress risks in the primary participant [45]. Food frequency questionnaires (FFQ) are administered to better understand the primary participant’s potential for herbicide exposure through dietary ingestion. Existing food frequency questionnaire instruments were not adequate to collect information relevant to herbicide exposures. Therefore, a specific food frequency questionnaire was developed and is used to collect information to allow estimation of dietary exposures to herbicides. The specific instrument is available from the study authors upon request. After delivery, the participant pregnancy questionnaire is administered to capture additional potential exposures that may have occurred during the pregnancy. Table 3 provides a list of questionnaire instruments administered and biospecimens collected with details regarding timing of administration. The Supplementary file contains actual questionnaires used.

**Table 3:** Timing of data collection, review, analysis, and compensation activities

Intervention	Phase 1 (Prenatal/Delivery)				
	**0–13 Week OB Visit**	**14–27 Week OB Visit**	**28–40 Week OB Visit**	**Delivery Visit**	**Post Delivery Visit**
Screen/Consent	X	X			
Urine Sample	X	X	X		
Buccal Sample	X	X	X	X	X
Maternal Pre-Conception Survey	X	X^a^	X^a^	X^a^	
Maternal Perceived Stress Survey (PSS)	X	X	X	X	
Maternal Food Frequency Questionnaire (FFQ)	X	X	X	X	
Paternal Pre-Conception Survey	X	X^a^	X^a^	X*	
Maternal Pregnancy Survey				X	X^a^
Child Health Surveys					
Chart Review	X	X	X	X	X
Participant Compensation	X	X	X	X	X

Legend: ^a^If not completed during initial visit in which assigned, questionnaires may be administered at subsequent visits

Study staff conduct medical chart reviews to collect perinatal, neonatal, and child developmental/ physiological data related to the pregnancy. A trained certified chart abstractor assesses all participant medical records to record final birth outcomes. Certification for chart abstraction is done locally at clinical sites based on operationalized study protocol under the direction of the site Principal Investigator. Study staff and chart abstractors are blinded to laboratory concentration assessments at all times during the study.

The study variables are used to identify risk factors, outcome measures and potential confounding factors. They include parent demographic variables such as age, race/ethnicity, educational attainment, income category to determine relationship to federal poverty level, zip code and county of residence, residential longitude and latitude values, family medical history, and month and year of birth; perinatal variables such as pregnancy history, conditions and complications of pregnancy, fetal development, conditions and complications of labor and delivery, pregnancy outcomes, pregnancy weight gain, pre-pregnancy and delivery BMI, and laboratory and imaging results; and neonatal variables such as conditions and complications of delivery and resuscitation, conditions and care of the newborn, APGAR scores, anthropometric measures (newborn weight, head circumference, height, percentiles, BMI), perinatal diagnoses and outcomes, discharge nutrition and feeding methods, growth patterns, laboratory and imaging results, and discharge status.

Individual research data will not be made available to the clinical care provider. Primary data will be collected via paper, electronic medical records, and online questionnaires and will be stored electronically in REDCap, SAS files, and excel spreadsheets on encrypted secure servers. Other data sources include outside lab data and USDA/EPA/USGS public-use datasets that will be stored in separate electronic files and merged with the primary datasets as needed. Data management and analysis will be performed by the study data coordination and analytics team. An outside study data monitor will evaluate compliance with study procedures and integrity of data collection and management.

### Study visits and specimen collection

The first prenatal specimen collection ideally occurs prior to 13 weeks’ gestation. If a participant is recruited after 13 weeks’ gestation, then first prenatal collection occurs as soon after enrollment as possible. At this study visit, site coordinators collect prenatal urine and buccal samples from the primary participant. If the primary participant consents to recruitment of the other biological parent, then the study coordinator also will approach the other biological parent for written informed consent. If given, they will collect buccal and urine specimens from the secondary participant, if consented. The study coordinator will email links to participants to complete pre-conception, food frequency, and stress questionnaires electronically. Study personnel conduct prenatal/medical chart reviews and contact participants for follow-up if needed.

The second prenatal collection occurs at the first prenatal visit that is between 14- and 27-weeks’ gestation and at least 4 weeks after the first prenatal collection while the third prenatal collection occurs at the first prenatal visit that is after 28 weeks’ gestation and at least 4 weeks after the second prenatal collection, if practicable. Collection of a specimen outside the ideal time frame is preferred over not collecting a specimen. Study team members collect a urine sample from the primary participant and email the primary participant links to food frequency and stress questionnaires. If applicable and not yet collected, they attempt to collect buccal and urine samples from the other consented biological parent and complete the pre-conception questionnaires with both participants if not previously administered. They conduct prenatal/medical chart reviews and contact participant for follow-up if needed.

Following delivery, study team members collect infant buccal specimens. If not previously collected, they also collect a third trimester urine specimen from the primary participant. The buccal specimen may be collected during the postpartum hospital stay or during an obstetrical follow-up visit up to 12 weeks after delivery. Study coordinators conduct prenatal/delivery medical chart reviews and contact participants for follow-up if needed. We employ multiple approaches for subject retention and engagement, including using social media, email, text message, study updates, and other methods that may be site specific. Our coordinator teams meet biweekly to discuss recruitment and engagement and share best practices.

### Exposure assessment

Coded urine and buccal samples collected at clinical locations will be transported to study site laboratories for processing per laboratory processing protocols and aliquoted into cryovials (as appropriate) and stored. Buccal and urine samples will be stored locally at ≤ -20 °C until they are shipped to the Indiana Bioservices biorepository in Indianapolis, Indiana, where they will be stored ≤ -70 °C. Samples will be batch shipped from Indiana Bioservices to an external analytic laboratory for completion of relevant assays. Details of the analytic methods utilized for biochemical concentration will be detailed in other manuscripts.

This study will utilize Geographical Information Systems and remote sensing data to develop herbicide exposure index estimates to complement biomonitoring results. An herbicide exposure index will be calculated using a buffer zone around a consenting participant’s residence. Using methods described in GIS-based exposure studies [46, 47], ArcGIS (ESRI Inc. Version 10.6.1) will be used to calculate the total land area within each the buffer zone for each crop type, which will be compiled based on USDA crop land use data [48]. The total area of each crop will be then multiplied by an average pesticide application rate estimated from data available from the USDA Chemical Use Program [49]. The total area of crop use land in each participant buffer zone (in km^2^) will be multiplied by the amount of pesticide application rate (in kg/km^2^) for each crop in order to estimate the total volume (in kg) of each pesticide applied within the buffer zone. An exposure index will be calculated for each pesticide expected to have been used. In addition, an aggregate exposure index will also be estimated. Herbicide exposure metrics will be utilized as an adjunct to biomarker analysis to evaluate relationships between geographic exposure and biomarker levels, as well as to refine associations between aggregate exposure and health outcomes.

### Outcomes

Study hypotheses relate to maternal and fetal reproductive outcomes that have been previously found to be associated with herbicide concentrations during pregnancy in human or animal studies. The primary outcome of the Heartland Study is gestational age at birth. Key human and animal studies supporting these hypotheses have been detailed in Tables 1 and 2. Maternal outcomes that will be considered are pregnancy loss, preterm birth, hypertensive disorders of pregnancy, and gestational diabetes. For purposes of the study, miscarriage is defined as the delivery of a liveborn fetus or a fetus who experienced fetal death for any cause before 20 + 0 weeks’ gestation. A miscarriage is considered early if delivery occurs at < 14 + 0 weeks and late if it occurs at ≥ 14 + 0 weeks’ gestation. Stillbirth is defined as fetal death before delivery after 20 weeks’ gestation [50]. Preterm birth is defined as delivery of a liveborn or stillborn infant for any reason between 22 + 0 and 36 + 6 weeks’ gestation [51]. Spontaneous preterm birth is defined as delivery that occurs after spontaneous onset of preterm labor or premature rupture of the membranes (PROM) or fetal membrane prolapse. Preterm labor is defined as spontaneous uterine contractions before membrane rupture, documented cervical change of at least 1 cm dilation or effacement during the admission, or dilation > 2 cm or effacement > 80% on admission for contractions. PROM is defined as spontaneous rupture of the membranes before the onset of contractions, regardless of subsequent labor augmentation or cesarean delivery. Indicated preterm birth is defined as delivery after induction or cesarean delivery between 20 + 0 and 36 + 6 weeks’ gestation for obstetric, maternal, or fetal indications. The indication for the delivery will be recorded. A birth is considered spontaneous unless documented otherwise. Term birth is defined as delivery of a liveborn or stillborn infant for any reason at ≥ 37 + 0 weeks’ gestation.

Hypertensive disorders of pregnancy include gestational hypertension, preeclampsia, eclampsia, and HELLP [hemolysis, elevated liver enzymes, and low platelet count] syndrome [52]. Gestational hypertension is defined as systolic blood pressure over 140 mmHg or diastolic blood pressure over 90 mmHg measured on at least two occasions 4 or more hours apart after 20 weeks’ gestation in a pregnant person that previously had normal blood pressure. Pre-eclampsia is gestational hypertension with proteinuria (300 mg or more in a 24-h urine collection, or other relevant clinical measurement). HELLP Syndrome and eclampsia are severe features of pre-eclampsia. HELLP Syndrome includes symptoms of Hemolysis, Elevated Liver Enzymes, and Low Platelet Count. Eclampsia is the manifestation of neurologic symptoms precipitated by hypertension. Diagnoses of these conditions made by a provider based on other clinical indications are also included.

Gestational diabetes is defined as a fasting plasma glucose ≥ 7.0 mmol/l (126 mg/ dl), a 2-h plasma glucose ≥ 11.1 mmol/l (200 mg/dl) following a 75 g oral glucose load, or a random plasma glucose ≥ 11.1 mmol/l (200 mg/ dl) in the presence of diabetes symptoms [53]. Additionally, gestational diabetes may be diagnosed clinically based on sequential screening with an abnormal 1 h glucose challenge test (50 g) followed by two abnormal values on a fasting 3-h glucose tolerance test, using appropriate cutoffs recommended by the American College of Obstetrics and Gynecology [54]. A participant is considered to have gestational diabetes if the criteria are documented or if a physician diagnosis of gestational diabetes is documented in the chart.

Primary fetal/newborn outcomes being evaluated are miscarriage, stillbirth, congenital anomalies/ malformations, fetal growth restriction, length of gestation, and birthweight. Secondary analyses may be performed to evaluate neonatal health status, including presence of respiratory distress syndrome, respiratory/ventilatory support, neonatal sepsis (confirmed/suspected, early/late onset), intraventricular hemorrhage, and infant discharge status. We are collecting buccal swabs which will be banked and plan to explore epigenetic changes associated with herbicide concentrations as well. We will plan the future epigenetic analyses based on techniques and methylated regions of interest that are cutting-edge and relevant at the time of those analyses. Specific plans for that future work are beyond the scope of this methods paper. The specific methods and hypotheses relating to the epigenetic analyses in the future will be detailed in papers arising from those analyses.

### Data management and storage

Data will be housed in a REDCap database. Direct entry into the database via secure links will be available for participants. Data collected at visits and chart abstraction will be entered by study staff. The Heartland Study REDCap database will be maintained at Indiana University and the data will undergo regular quality control checks and cleaning. When ready for analysis, the data will be securely transmitted to the study statistical analysis team.

A Data Coordinating and Analytics Core, housed at Indiana University School of Medicine, is responsible for data monitoring and quality control. Regular reports and data queries are made to study sites to ensure data accuracy. A study monitor with regulatory experience is performing study data monitoring and regulatory compliance regularly throughout the study and making site visits for document and data verification.

## Statistical analysis

The Heartland Study is designed as a prospective cohort study with target recruitment of 2,000 pregnancies. Anticipated loss to follow-up is estimated at conservatively estimated at 20%, yielding approximately 1,800 analyzable maternal-infant dyads for the longer-term Phase 2 portion of the study. Based on prevalence estimates reported in prior studies [55–60], it is estimated that this will yield (all approximated) 180 preterm births, 288 cases of hypertensive disorders of pregnancy, 126 cases of gestational diabetes, 90 cases of fetal growth restriction, 54 cases of congenital anomalies/malformations, and 11 cases of stillbirth. The detectable R^2^ attributed to the herbicide exposure variable of interest from a multiple regression model fit to the primary outcome (gestational age) adjusting for 5 covariates (with combined R^2^ of 0.20 by covariates themselves), with 80% power and a type I error of 0.05, is 0.003. The detectable odds ratios for presence of secondary outcomes associated with a one standard deviation increase in the herbicide exposure variable of interest range between 1.3 and 1.7 (excluding stillbirth), with type I error set at 0.0083 to keep familywise error at 0.05 for secondary outcomes [61]. Thus, the study is powered to detect several meaningful associations between herbicide exposures of interest and pregnancy outcomes.

Descriptive statistics, including appropriate parametric and semiparametric methods, will be used for continuous, binary, ordinal, and polytomous variables to describe characteristics of the cohort, exposures, and outcomes. We will examine the concentration data for appropriateness of cutoff values (quartiles, quintiles, high/low, etc.) or use as a continuous variable. Examples of statistical modeling approaches that we intend to utilize include least-squares linear regression, which will be used to describe the unadjusted associations between continuous outcomes (e.g., length of gestation or birthweight) and longitudinal exposure variable; and logistic regression, which will be used to describe the unadjusted association between a dichotomous outcome (e.g., diagnosis of pre-eclampsia or gestational diabetes, presence of congenital anomaly) and longitudinal exposure variables. Multivariate models will also be fit using suitable stratification and modeling adjustments for confounding and assessments of effect modification to evaluate associations between each outcome and multiple longitudinal exposures using methods appropriate to the structure of the data and analyses [62]. We will begin with simple multiple logistic regression models but will adjust to other appropriate models based on the data. To evaluate for the presence of exposure misclassification, an exposure matrix variable utilizing both biomarker and geospatially derived exposure estimates will be modeled with respect to each outcome [63].

Confounding variables have been determined a priori based on prior studies. Potential confounding factors to be considered are maternal age [64, 65], country of birth [66, 67], race/ethnicity [68, 69], height and preconception weight (including Body Mass Index) [70, 71], pregnancy weight gain [72, 73], marital status [64, 74], employment status [75, 76], socioeconomic status [77, 78], education level [78, 79], smoking status [80, 81], insurance coverage [82–84], alcohol use [85, 86], drug use, caffeine use [87], history of fetal loss and gestational age at loss [88], parity [89, 90], prenatal care [91], and rurality [92, 93]. As we will have residential address information, we will be able to explore the impact of location-based markers of social determinants of health such as distance to health care facilities, area deprivation, green space, and others.

## Challenges and anticipated responses

While the Heartland Study is anticipated to be a relatively large cohort of at least 2000 pregnancy dyads, it may still be underpowered to identify associations between herbicides and some rare outcomes, such as stillbirth and specific congenital anomalies. Aggregation of related outcomes may be used to improve power where appropriate. For example, it may be appropriate to evaluate fetal death as an aggregate variable including stillbirth and miscarriage, which occur on a spectrum. Related congenital anomalies may be aggregated to achieve statistical power if appropriate.

This study is poised to make an important contribution to the existing knowledge about herbicide concentration. There may be limitations in the ability to detect important seasonal variations in concentration and sensitive windows for exposure, although recurrent urine samples are being collected throughout pregnancy and aggregate batches by phases of the growing season will be evaluated. The study will utilize the latest advancements in analytic techniques for urine biomarker analysis, providing an objective assessment of concentration. Multi-exposure modeling will be used to examine the combined effects of herbicide exposure. Geographic estimates of exposure will be used to augment biomarker data and allow for a more multidimensional exposure assessment. Through combined application of the techniques, the Heartland Study will fill important gaps in knowledge about herbicide exposures in the Midwest.

### Supplementary Information

**Additional file 1.**

## Data Availability

At the end of the study, we plan to deposit data into a public data repository for use by other researchers as needed. Until data are deposited, we will entertain request for data sharing and collaboration.

## Abbreviations

BMI: Body Mass Index
EDD: Estimated Due Date
EGA: Estimated Gestational Age
EPA: United States Environmental Protection Agency
FFQ: Food Frequency Questionnaire
HELLP: Hemolysis, Elevated Liver Enzymes, Low Platelet Count
LMP: Last Menstrual Period
PSS: Perceived Stress Scale
PROM: Premature Rupture of Membranes
US: Ultrasound
USDA: United States Department of Agriculture
USGS: United States Geological Service
