# Supplementary material for: Perinatal health effects of herbicides exposures in the United States: the Heartland Study, a Midwestern birth cohort study
Source: BMC Public Health. 2023 Nov 22;23:2308. doi: 10.1186/s12889-023-17171-9 (PMC10664386; doi:10.1186/s12889-023-17171-9)
Supplement: Supplementary file 1 — Additional file 1. [file 12889_2023_17171_MOESM1_ESM.pdf]

REPRODUCTIVE HISTORY

1) Approximately how old were you at the time of your first menstrual period?

_________Years of Age

2) Do you recall where you lived at the time of your first menstrual period?

☐ Yes
☐ No

If yes, where did you live at the time of your first menstrual period? *Complete as much as known*

Country: ___________________________________

State/Territory: _____________________________

City: ______________________________________

County: ____________________________________

If yes, what best describes the geographical area where you lived at the time of your first menstrual period?

☐ Rural, mostly farmland, agricultural fields
☐ Rural, little or no farming nearby
☐ Suburban, close to city, outskirts, neighborhoods
☐ Urban, inner city, downtown, metropolitan area
☐ Don't Know

If yes, do you recall if you lived on a farm at the time of your first menstrual period?

☐ Yes
☐ No
☐ Not Sure

3) Was your current pregnancy a planned pregnancy?

☐ Yes
☐ No

If yes, how long did you try before you became pregnant?

_________Years _________Months

4) Is your current pregnancy your first pregnancy?

☐ Yes
☐ No

If no, did you become pregnant less than 18 months from the end of your last pregnancy?

☐ Yes
☐ No

5) Have you ever been told by a doctor that you have female or reproductive health problems?

☐ Yes
☐ No
☐ Not Sure

If yes, did you have any of the following health problems?

☐ Uterine Fibroids (non-cancerous tumors that grow in and around your uterus)
☐ Polycystic Ovarian Syndrome (PCOS) (hormonal problems that can cause cysts to grow on your ovaries)
☐ Endometriosis (tissue that normally lines the inside of the uterus that also grows outside the uterus)
☐ Abnormal Uterus (e.g. heart shaped uterus, small uterus, double uterus, tipped uterus, etc.)
☐ Other female or reproductive health problem(s)

Please specify ______________________________

____

6) What is the total number of times that you have been pregnant including this pregnancy and any miscarriages, stillbirths and abortions?

_________Total Pregnancies

7) With your current pregnancy, did you take any herbal supplements to help you become pregnant?

☐ Yes
☐ No

8) With your current pregnancy, did you make any dietary changes to help you become pregnant?

☐ Yes
☐ No

9) With your current pregnancy, did you have difficulty becoming pregnant?

☐ Yes
☐ No

10) Have you taken fertility drugs to help you become pregnant such as Clomid, Pergonal, HCG (Pregnyl, Novarel, Ovidrel) , Lupron, Metrodin or other?

☐ Yes, with current pregnancy
☐ Yes, with prior pregnancies
☐ No

REPRODUCTIVE HISTORY continued

11) Have you used in-vitro fertilization (IVF) to help you to become pregnant? *Check all that apply*
   - ☐ Yes, with current pregnancy
   - ☐ Yes, with prior pregnancies
   - ☐ No

   If yes with your current pregnancy, how many IVF cycles did you have before you became pregnant:

   _________IVF cycles with current pregnancy

12) With your current pregnancy, how much did you weigh just before you became pregnant?

   _________Pounds

13) How tall are you?

   ________Feet _________Inches

FAMILY MEDICAL HISTORY

1) Have you, or your biological family members, ever been told that you have or had any of the following health conditions?

<u>*Definitions:*</u> ***Self*** *refers to yourself;* ***Immediate family member*** *refers to your biological parents, brothers, sisters, and children;* ***Other blood relatives*** *refers to your biological grandparents, aunts, uncles, cousins, half siblings where you share a parent, etc.*

Hypothyroidism (Underactive Thyroid)
- ☐ No-one
- ☐ Self
- ☐ Immediate Family Member
- ☐ Other Blood Relative

Hyperthyroidism (Overactive Thyroid)
- ☐ No-one
- ☐ Self
- ☐ Immediate Family Member
- ☐ Other Blood Relative

Diabetes – Type 1
- ☐ No-one
- ☐ Self
- ☐ Immediate Family Member
- ☐ Other Blood Relative

Diabetes – Type 2
- ☐ No-one
- ☐ Self
- ☐ Immediate Family Member
- ☐ Other Blood Relative

Gestational Diabetes
- ☐ No-one
- ☐ Self
- ☐ Immediate Family Member
- ☐ Other Blood Relative

Crohn's Disease
- ☐ No-one
- ☐ Self
- ☐ Immediate Family Member
- ☐ Other Blood Relative

Irritable Bowel Syndrome (IBS)
- ☐ No-one
- ☐ Self
- ☐ Immediate Family Member
- ☐ Other Blood Relative

Arthritis (e.g. Osteoarthritis, Rheumatoid Arthritis)
- ☐ No-one
- ☐ Self
- ☐ Immediate Family Member
- ☐ Other Blood Relative

Lupus
- ☐ No-one
- ☐ Self
- ☐ Immediate Family Member
- ☐ Other Blood Relative

Fibromyalgia
- ☐ No-one
- ☐ Self
- ☐ Immediate Family Member
- ☐ Other Blood Relative

Chronic Fatigue Syndrome
- ☐ No-one
- ☐ Self
- ☐ Immediate Family Member
- ☐ Other Blood Relative

Asthma
- ☐ No-one
- ☐ Self
- ☐ Immediate Family Member
- ☐ Other Blood Relative

FAMILY MEDICAL HISTORY continued

Chronic Obstructive Pulmonary Disease (COPD)
- ☐ No-one
- ☐ Self
- ☐ Immediate Family Member
- ☐ Other Blood Relative

High Cholesterol
- ☐ No-one
- ☐ Self
- ☐ Immediate Family Member
- ☐ Other Blood Relative

Heart Disease
- ☐ No-one
- ☐ Self
- ☐ Immediate Family Member
- ☐ Other Blood Relative

Hypertension (High Blood Pressure)
- ☐ No-one
- ☐ Self
- ☐ Immediate Family Member
- ☐ Other Blood Relative

Pregnancy Induced Hypertension
- ☐ No-one
- ☐ Self
- ☐ Immediate Family Member
- ☐ Other Blood Relative

Stroke, Transient Ischemic Attack (TIA)
- ☐ No-one
- ☐ Self
- ☐ Immediate Family Member
- ☐ Other Blood Relative

Depression, Anxiety
- ☐ No-one
- ☐ Self
- ☐ Immediate Family Member
- ☐ Other Blood Relative

Memory Loss, Confusion
- ☐ No-one
- ☐ Self
- ☐ Immediate Family Member
- ☐ Other Blood Relative

Learning Disability such as Dyslexia, etc.
- ☐ No-one
- ☐ Self
- ☐ Immediate Family Member
- ☐ Other Blood Relative

Seizures, Epilepsy
- ☐ No-one
- ☐ Self
- ☐ Immediate Family Member
- ☐ Other Blood Relative

Hyperactivity, ADHD, ADD
- ☐ No-one
- ☐ Self
- ☐ Immediate Family Member
- ☐ Other Blood Relative

Autism
- ☐ No-one
- ☐ Self
- ☐ Immediate Family Member
- ☐ Other Blood Relative

Lymphoma or Hodgkin's disease
- ☐ No-one
- ☐ Self
- ☐ Immediate Family Member
- ☐ Other Blood Relative

Other Cancer(s); please specify below
- ☐ No-one
- ☐ Self
- ☐ Immediate Family Member
- ☐ Other Blood Relative

Birth Defect(s); please specify below
- ☐ No-one
- ☐ Self
- ☐ Immediate Family Member
- ☐ Other Blood Relative

Other Condition; please specify below
- ☐ No-one
- ☐ Self
- ☐ Immediate Family Member
- ☐ Other Blood Relative

If other cancer(s), birth defect(s) or other condition(s) checked above, please describe below:

Other Cancer(s): ______________________________
____

Birth Defect(s): ______________________________
____

Other Condition(s): ____________________________
____

RESIDENTIAL HISTORY

1) Where do you currently live?

Street Address: ______________________________

City: ______________________________

State: ______________________________

Zip Code: ______________________________

Approximately, when did you move to where you currently live?

Date: ______________(mm/dd/yyyy)

2) Is the place where you currently live located on a farm?

☐ Yes
☐ No
☐ Don't Know

3) What best describes the geographical area where you currently live?

☐ Rural, mostly farmland, agricultural fields
☐ Rural, little or no farming nearby
☐ Suburban, close to city, outskirts, neighborhoods
☐ Urban, inner city, downtown, metropolitan area
☐ Don't Know

4) Is the place where you currently live located within 1 mile of any of the following?

☐ Golf Course
☐ Park or Recreational Area
☐ School
☐ Soccer, Football, Baseball Fields
☐ Cemetery
☐ None of the Above

5) To the best of your knowledge, was the home where you currently live, built before 1978?

☐ Yes
☐ No
☐ Don't Know

6) Has the home where you currently live ever been treated or sprayed for insects or rodents?

☐ Yes
☐ No
☐ Don't Know

7) Where did you live prior to your current location?

Street Address: ______________________________

City: ______________________________

State: ______________________________

Zip Code: ______________________________

Approximately, when did you move to this prior location?

Date: ______________ (mm/dd/yyyy)

8) Was the place that you lived prior to your current location on a farm?

☐ Yes
☐ No
☐ Don't Know

9) What best describes the geographical area where you lived prior to your current location?

☐ Rural, mostly farmland, agricultural fields
☐ Rural, little or no farming nearby
☐ Suburban, close to city, outskirts, neighborhoods
☐ Urban, inner city, downtown, metropolitan area
☐ Don't Know

10) Is the place where you lived prior to your current location within 1 mile of any of the following?

☐ Golf Course
☐ Park or Recreational Area
☐ School
☐ Soccer, Football, Baseball Fields
☐ Cemetery
☐ None of the Above

11) To the best of your knowledge, was the home where you lived prior, built before 1978?

☐ Yes
☐ No
☐ Don't Know

12) Was the home where you lived prior ever treated or sprayed for insects or rodents?

☐ Yes
☐ No
☐ Don't Know

RESIDENTIAL HISTORY (continued)

13) Do you know the place where you were born?
- ☐ Yes
- ☐ No

If yes, please indicate the place where you were born:
Complete as much as you can:

Country: ______________________________

State/Territory: ______________________________

City: ______________________________

County: ______________________________

If yes, approximately how many years did you live in the place where you were born?

_________Years

If yes, how would you best describe the geographical area where your home in your birthplace was located?
- ☐ Rural, mostly farmland, agricultural fields
- ☐ Rural, little or no farming nearby
- ☐ Suburban, close to city, outskirts, neighborhoods
- ☐ Urban, inner city, downtown, metropolitan area
- ☐ Don't Know

If yes, was your home in your birthplace located on a farm?
- ☐ Yes
- ☐ No
- ☐ Don't Know

If yes, was your home in your birthplace located within one mile of any of the following?
- ☐ Golf Course
- ☐ Park or Recreational Area
- ☐ School
- ☐ Soccer, Football, Baseball Fields
- ☐ Cemetery
- ☐ None of the Above

14) At any time during your childhood, were any of the homes in which you lived found to have lead?
- ☐ Yes
- ☐ No
- ☐ Don't Know

WATER SOURCES

1) During the 3 months before your pregnancy, where did your residential water come from?
- ☐ Well
- ☐ City, Public, or Private Water Company
- ☐ Don't Know

If city, public or private water selected, please list your water provider: ______________________________

2) During the 3 months before your pregnancy, did you use filtered water in your home such as water faucet filtration, refrigerator water filtration, water softener, reverse osmosis, water dispenser or cooler?
- ☐ Yes
- ☐ No
- ☐ Don't Know

3) During the 3 months before your pregnancy, what were your drinking water sources?
- ☐ Unfiltered Tap Water

  If yes, how many 8 ounces glasses of tap water do you drink per day? _______Glasses per day
- ☐ Filtered Water

  If yes, how many 8 ounces glasses of filtered water do you drink per day? _______Glasses per day
- ☐ Bottled Water

  If yes, how many 8 ounces glasses of bottled water do you drink per day? _______Glasses per day

Bottled Water Chart
33.8 fluid ounces (1 liter) = 4 glasses
20 fluid ounces (0.591 liter, 591 mL) = 2½ glasses
16.9 fluid ounces (0.5 liter, 500 mL) = 2 glasses
12 fluid ounces (0.355 liter, 355 mL) = 1½ glasses
11.5 fluid ounces (0.330 liter, 330 mL) = 1½ glasses

SUBSTANCE USE

This information is being collected for ***research purposes only*** and will not be put into your medical record or shared with your provider.

1) What best describes your use of alcohol?
   - ☐ Never Used
   - ☐ Currently Used
   - ☐ Formerly Used

   If currently or formerly used, did you drink alcohol during the 3 months before your pregnancy?
   - ☐ Yes
   - ☐ No

   If yes, how many days per week did you drink?
   - ☐ Less than 1 day per week
   - ☐ Greater than or equal to 1 day per week

   If yes, on average, how many drinks did you drink per week?

   _________Drinks per week

2) What best describes your use of tobacco?
   - ☐ Never Used
   - ☐ Currently Used
   - ☐ Formerly Used

   If currently or formerly used, did you smoke tobacco during the 3 months before your pregnancy?
   - ☐ Yes
   - ☐ No
   - ☐ Don't Know

   If yes, on average, how many did you smoke per day?

   _________Smoked per day

3) During the 3 months before your pregnancy, did anyone in your household, other than yourself, smoke tobacco?
   - ☐ Yes
   - ☐ No
   - ☐ Don't Know

4) What best describes your use of e-cigarettes or vaping?
   - ☐ Never Used
   - ☐ Currently Use
   - ☐ Formerly Used

   If currently or formerly used, did you vape or use e-cigarettes during the 3 months before your pregnancy?
   - ☐ Yes
   - ☐ No
   - ☐ Don't Know

   If yes, on average how many times did you vape or e-cig per day?

   _________Vapes or e-cigs per day

5) During the 3 months before your pregnancy, did anyone in your household, other than yourself, vape or e-cig?
   - ☐ Yes
   - ☐ No
   - ☐ Don't Know

6) What best describes your use of marijuana (THC), Cannabinoids, or CBD oil?
   - ☐ Never Used
   - ☐ Currently Use
   - ☐ Formerly Used

   If currently or formerly use, did you use marijuana (THC), cannabinoids, or CBD oil during the 3 months before your pregnancy?
   - ☐ Yes
   - ☐ No
   - ☐ Don't Know

7) What best describes your use of prescribed methadone, Buprenorphine, Suboxone or subutex?
   - ☐ Never Used
   - ☐ Currently Use
   - ☐ Formerly Used

   If currently or formerly used, did you use prescribed methadone, buprenorphine, Suboxone, or subutex during the 3 months before your pregnancy?
   - ☐ Yes
   - ☐ No
   - ☐ Don't Know

8) What best describes your use of other prescription drugs not prescribed to you?
   - ☐ Never Used
   - ☐ Currently Use
   - ☐ Formerly Used

   If you currently or formerly used, did you use other prescription drugs not prescribed to you during the 3 months before your pregnancy?
   - ☐ Yes
   - ☐ No
   - ☐ Don't Know

SUBSTANCE USE continued

This information is being collected for ***research purposes only,*** and will not be put into your medical record or shared with your provider.

9) What best describes your use of other drugs such as heroin, cocaine, amphetamines, etc.?
   - ☐ Never Used
   - ☐ Currently Use
   - ☐ Formerly Used

If you currently or formerly used, did you use other drugs such as heroin, cocaine, amphetamines, etc. during the 3 months before your pregnancy?
   - ☐ Yes
   - ☐ No
   - ☐ Don't Know

SPORTS, HOBBIES, RECREATIONAL ACTIVITIES, TRAVEL

1) During the 3 months before your pregnancy, did you do any of the following sports, hobbies, crafts, recreational activities?

Water sports such as swimming, skiing, tubing, surfing, etc.
   - ☐ Never/Rarely
   - ☐ Daily
   - ☐ Weekly
   - ☐ Monthly

Underwater sports such as snorkeling, scuba diving, etc.
   - ☐ Never/Rarely
   - ☐ Daily
   - ☐ Weekly
   - ☐ Monthly

Boating, sailing, canoeing, rafting, kayaking, etc.
   - ☐ Never/Rarely
   - ☐ Daily
   - ☐ Weekly
   - ☐ Monthly

Camping, hiking, fishing, hunting, etc.
   - ☐ Never/Rarely
   - ☐ Daily
   - ☐ Weekly
   - ☐ Monthly

Biking, running, jogging, walking, etc.
   - ☐ Never/Rarely
   - ☐ Daily
   - ☐ Weekly
   - ☐ Monthly

Golfing, driving range
   - ☐ Never/Rarely
   - ☐ Daily
   - ☐ Weekly
   - ☐ Monthly

Rock or mountain climbing, sky or cliff diving, parasailing
   - ☐ Never/Rarely
   - ☐ Daily
   - ☐ Weekly
   - ☐ Monthly

Winter sports such as skiing, snowboarding, snowmobiling
   - ☐ Never/Rarely
   - ☐ Daily
   - ☐ Weekly
   - ☐ Monthly

Attended/played outdoor field sports (soccer, football, etc.)
   - ☐ Never/Rarely
   - ☐ Daily
   - ☐ Weekly
   - ☐ Monthly

Sitting in saunas or hot tubs
   - ☐ Never/Rarely
   - ☐ Daily
   - ☐ Weekly
   - ☐ Monthly

Jewelry/pottery making, stained glass, oil painting, ceramics
   - ☐ Never/Rarely
   - ☐ Daily
   - ☐ Weekly
   - ☐ Monthly

Other, please specify: ______________________________
   - ☐ Never/Rarely
   - ☐ Daily
   - ☐ Weekly
   - ☐ Monthly

SPORTS, HOBBIES, RECREATIONAL ACTIVITIES, TRAVEL continued

2) During the 3 months before your pregnancy, did you travel outside of the U.S.?
- ☐Yes
- ☐No
- ☐Don't Know

If yes, how many times did you travel outside of the U.S.?

_________Times

If yes, what country(s) did you visit?

____

If yes, were any of the places that you visited near agriculture fields or farmland?
- ☐Yes
- ☐No
- ☐Don't Know

If yes, were you outside of the U.S. on any one trip for greater than 1 week?
- ☐Yes
- ☐No
- ☐Don't Know

If yes, approximately how many weeks were you outside of the U.S.?

________Weeks

HOME RELATED ACTIVITIES

Instructions: Please check how frequency you used conventional chemicals (products that contain chemicals) and eco-friendly (products that contain natural ingredients; also referred to as biochemical, biodegradable, environmentally friendly or green products).

1) During the 3 months before your pregnancy, how many times did you handle, mix, or apply chemicals or fertilizers while gardening, farming, landscaping, lawn care, or other activities?

To manage or kill insects:

Conventional	***Eco-Friendly***
☐ Never/Rarely	☐ Never/Rarely
☐1-2 times	☐ 1-2 times
☐ 3-5 times	☐ 3-5 times
☐ 5 times or more	☐5 times or more

To manage or kill weeds:

Conventional	***Eco-Friendly***
☐ Never/Rarely	☐ Never/Rarely
☐ 1-2 times	☐ 1-2 times
☐ 3-5 times	☐ 3-5 times
☐5 times or more	☐5 times or more

To control plant molds or fungal diseases:

Conventional	***Eco-Friendly***
☐ Never/Rarely	☐ Never/Rarely
☐ 1-2 times	☐ 1-2 times
☐ 3-5 times	☐ 3-5 times
☐5 times or more	☐5 times or more

To fertilize your lawns, gardens, etc.:

Conventional	***Eco-Friendly***
☐ Never/Rarely	☐ Never/Rarely
☐ 1-2 times	☐ 1-2 times
☐ 3-5 times	☐ 3-5 times
☐5 times or more	☐ 5 times or more

2) If known, please list the chemicals you referred to in your responses above:

Used to manage or kill insects:

____

Used to manager or kill weeds:

____

Used to control plant molds or fungal diseases:

____

Used to fertilize your lawns, gardens, etc.:

____

HOUSEHOLD CLEANING INFORMATION

1) During the 3 months before your pregnancy, how frequently did you use the following household cleaners?

Sanitizers, disinfectants (e.g. Lysol, Clorox, etc.):

Conventional	***Eco-Friendly***
☐ Never/Rarely	☐ Never/Rarely
☐ Daily	☐ Daily
☐ Weekly	☐ Weekly
☐ Monthly	☐ Monthly

Chlorine Bleach:

Conventional	***Eco-Friendly***
☐ Never/Rarely	☐ Never/Rarely
☐ Daily	☐ Daily
☐ Weekly	☐ Weekly
☐ Monthly	☐ Monthly

Detergents or Stain Removers:

Conventional	***Eco-Friendly***
☐ Never/Rarely	☐ Never/Rarely
☐ Daily	☐ Daily
☐ Weekly	☐ Weekly
☐ Monthly	☐ Monthly

Carpet or Upholstery Cleaners

Conventional	***Eco-Friendly***
☐ Never/Rarely	☐ Never/Rarely
☐ Daily	☐ Daily
☐ Weekly	☐ Weekly
☐ Monthly	☐ Monthly

Furniture or Floor Polish

Conventional	***Eco-Friendly***
☐ Never/Rarely	☐ Never/Rarely
☐ Daily	☐ Daily
☐ Weekly	☐ Weekly
☐ Monthly	☐ Monthly

Oven Cleaners

Conventional	***Eco-Friendly***
☐ Never/Rarely	☐ Never/Rarely
☐ Daily	☐ Daily
☐ Weekly	☐ Weekly
☐ Monthly	☐ Monthly

Air or Carpet Fresheners/Deodorizers

Conventional	***Eco-Friendly***
☐ Never/Rarely	☐ Never/Rarely
☐ Daily	☐ Daily
☐ Weekly	☐ Weekly
☐ Monthly	☐ Monthly

Dry Cleaning Chemicals:

Conventional	***Eco-Friendly***
☐ Never/Rarely	☐ Never/Rarely
☐ Daily	☐ Daily
☐ Weekly	☐ Weekly
☐ Monthly	☐ Monthly

2) During the 3 months before your pregnancy, did you clean homes, launder clothes or provide professional cleaning services other than for your own household?
   - ☐ Yes
   - ☐ No

If yes, how frequently did you _clean homes_?
- ☐ Never/Rarely
- ☐ Daily
- ☐ Weekly
- ☐ Monthly

If yes, how frequently did you _launder clothes_?
- ☐ Never/Rarely
- ☐ Daily
- ☐ Weekly
- ☐ Monthly

If yes, how frequently did you _provide professional cleaning services_?
- ☐ Never/Rarely
- ☐ Daily
- ☐ Weekly
- ☐ Monthly

OCCUPATION AND WORK ENVIRONMENT

1) During the 3 months before your pregnancy, what was your work situation/status? *Check all that apply*
   - ☐ Employed
   - ☐ Intern/Apprentice
   - ☐ Unemployed/Laid Off
   - ☐ Stay at home (do not work)
   - ☐ Rehabilitation/Disabled
   - ☐ Other, please describe below:

   ____

2) During the 3 months before your pregnancy, did you work outside your home?
   - ☐ Yes
   - ☐ No

   If yes, how many hours per week did you work outside your home?

   _________Hours per week

   If yes, was your workplace located on or close to a farm?
   - ☐ Yes
   - ☐ No
   - ☐ Don't Know

   If yes, describe the geographical area where your workplace was located:
   - ☐ Rural, mostly farmland, agricultural fields
   - ☐ Rural, little or no farming nearby
   - ☐ Suburban, close to city, outskirts, neighborhoods
   - ☐ Urban, inner city, downtown, metropolitan area
   - ☐ Don't Know

   If yes, was your workplace located within 1 mile of any of the following?
   - ☐ Golf Course
   - ☐ Park or Recreational Area
   - ☐ School
   - ☐ Soccer, Football, Baseball Fields
   - ☐ Cemetery

   If yes, please describe the type of work you carried out as accurately as possible (e.g. hospital department for children with cancer, body shop at a garage for diesel vehicles, farming with dairy cows, etc.):

   ____

   ____

3) During the 3 months before your pregnancy, did you mix, handle, or apply any of the following chemicals for work?

   Petroleum plastics (e.g. propylene or butylene glycol, etc.)
   - ☐ Yes
   - ☐ No
   - ☐ Don't Know

   Chrome, arsenic, cadmium, lead, mercury
   - ☐ Yes
   - ☐ No
   - ☐ Don't Know

   Gasoline or exhaust other than when filling gasoline in your car
   - ☐ Yes
   - ☐ No
   - ☐ Don't Know

   Pesticides such as weed killer, insecticides, fungicides
   - ☐ Yes
   - ☐ No
   - ☐ Don't Know

   Laughing gas or other anesthetic gases other than as a patient
   - ☐ Yes
   - ☐ No
   - ☐ Don't Know

   Mouse or rat poisons
   - ☐ Yes
   - ☐ No
   - ☐ Don't Know

   Chemotherapeutic substances/treatment other than as a patient
   - ☐ Yes
   - ☐ No
   - ☐ Don't Know

   Oil/water-based latex paints, paint thinners, lacquer, glue remover
   - ☐ Yes
   - ☐ No
   - ☐ Don't Know

   Industrial dyes/ink, photographic chemicals, fixatives, developers
   - ☐ Yes
   - ☐ No
   - ☐ Don't Know

   Motor oil, lubrication oil or other types of oils
   - ☐ Yes
   - ☐ No
   - ☐ Don't Know

OCCUPATION AND WORK ENVIRONMENT continued

Welding, soldering, etc.
- ☐ Yes
- ☐ No
- ☐ Don't Know

Formalin/formaldehyde
- ☐ Yes
- ☐ No
- ☐ Don't Know

Dry cleaning fluids (i.e. PERC, etc.)
- ☐ Yes
- ☐ No
- ☐ Don't Know

Foam, fiberglass, blown-in insulation, etc.
- ☐ Yes
- ☐ No
- ☐ Don't Know

Other, please specify: ________________________________
- ☐ Yes
- ☐ No
- ☐ Don't Know

MILITARY

1) At any time, have you ever served in the military including Reserve or National Guard?
   - ☐ Yes
   - ☐ No
   - ☐ Don't Know

   If yes, in what countries were you stationed?

   ____

   ____

   If yes, what is your current status?
   - ☐ Active Duty
   - ☐ Reserve
   - ☐ National Guard
   - ☐ Veteran

If yes, to the best of your knowledge, were you exposed to any of the following chemicals?
- ☐ Agent Orange or other herbicides used to destroy foliage/crops
- ☐ Pesticides used to repel or destroy insects and pathogens
- ☐ Industrial Chemicals found on some military bases
- ☐ PCBs typically used as coolant and insulating fluid
- ☐ Depleted Uranium used in military tank armor and bullets
- ☐ PFAs typically found in products including fire-fighting foams
- ☐ Shrapnel/toxic metals that remained in your body after injury
- ☐ Sand, Dust, and Particulates
- ☐ Sulfur Fire
- ☐ Ionizing Radiation, X Rays, Nuclear fallout
- ☐ Chromium
- ☐ Burn Pits used to dispose of military site waste
- ☐ Not that I'm aware of
- ☐ Other, please specify below:

____

____

COVID-19

1) Before your pregnancy, did you suspect that you or someone in your household had COVID-19?
   - ☐ Yes
   - ☐ No
   - ☐ Possibly
   - ☐ Don't Know

2) Before your pregnancy, did you have any symptoms(e.g. fever, dry cough, sore throat, difficulty breathing for shortness of breath,chest pain or pressure, fatigue, body or muscle aches, loss of appetite, loss of taste or smell,headache, chills or repeated shaking, nausea or vomiting,diarrhea or stomach problems)?
   - ☐ Yes
   - ☐ No
   - ☐ Unsure

   If yes, did you take medications to treat your symptoms?
   - ☐ Yes
   - ☐ No

   If yes, what medications did you take?

   ____

   ____

3) Before your pregnancy, has anyone in your household other than yourself tested positive for COVID-19?
   - ☐ Yes
   - ☐ No
   - ☐ Unsure

4) Before your pregnancy, were you ever tested for COVID-19?
   - ☐ Yes
   - ☐ No

   If yes, did you ever test positive for COVID-19?
   - ☐ Yes
   - ☐ No

   If yes, when did you test positive for COVID-19?

   Date _______________ (mm/dd/yyyy)

   Date _______________ (mm/dd/yyyy)

5) Did you self-quarantine due to COVID-19?
   - ☐ Yes
   - ☐ No

   If yes, how many days were you in self-quarantine?

   _________Days

6) Were you ever seen by a doctor or an ER visit due to COVID-19 (confirmed or suspected)?
   - ☐ Yes
   - ☐ No

7) Were you admitted to the hospital for treatment?
   - ☐ Yes
   - ☐ No

   If yes, approximately when were you in the hospital?

   Date admitted ________________ (mm/dd/yyyy)

   Date discharged _______________ (mm/dd/yyyy)

   If yes, were you put on a ventilator to help you breathe?
   - ☐ Yes
   - ☐ No

   If yes, number of days on ventilator?

   _________Days

   If yes, once released from the hospital did you self-quarantine?
   - ☐ Yes
   - ☐ No

   If yes, number of days in self-quarantine?

   ________Days

DEMOGRAPHICS

1) What is your current marital status?
   - ☐ Never Married, Living Alone
   - ☐ Never Married, Living with Significant Other
   - ☐ Married
   - ☐ Separated
   - ☐ Divorced
   - ☐ Widowed

2) What is your total combined annual gross (before taxes) household income?

   $______________ Annual Gross Income

3) How many family members, including yourself, are living in your household?

   _________ People

4) How many people including yourself are dependent on your annual gross household income?

   _________ People

5) How many children under the age of 18 live in your household?

   _________ Children

6) Are you currently receiving any type of financial assistance such as income assistance, disability assistance, housing assistance, utility assistance, childcare assistance, medical or healthcare assistance, prescription drug assistance, or other type of financial assistance?
   - ☐ Yes
   - ☐ No
   - ☐ Don't Know

7) How much did you weigh just before delivery?

   _________ Pounds

FAMILY MEDICAL HISTORY

1) During your pregnancy, were you told by a doctor that you have any of the following conditions?

Hypothyroidism (Underactive Thyroid)
- ☐ Yes
- ☐ No

Hyperthyroidism (Overactive Thyroid)
- ☐ Yes
- ☐ No

Diabetes – Type 1
- ☐ Yes
- ☐ No

Diabetes – Type 2
- ☐ Yes
- ☐ No

Gestational Diabetes
- ☐ Yes
- ☐ No

Crohn's Disease
- ☐ Yes
- ☐ No

Irritable Bowel Syndrome (IBS)
- ☐ Yes
- ☐ No

Arthritis (e.g. Osteoarthritis, Rheumatoid Arthritis)
- ☐ Yes
- ☐ No

Lupus
- ☐ Yes
- ☐ No

Fibromyalgia
- ☐ Yes
- ☐ No

Chronic Fatigue Syndrome
- ☐ Yes
- ☐ No

Asthma
- ☐ Yes
- ☐ No

Chronic Obstructive Pulmonary Disease (COPD)
- ☐ Yes
- ☐ No

High Cholesterol
- ☐ Yes
- ☐ No

Heart Disease
- ☐ Yes
- ☐ No

Hypertension (High Blood Pressure)
- ☐ Yes
- ☐ No

Pregnancy Induced Hypertension
- ☐ Yes
- ☐ No

Stroke, Transient Ischemic Attack (TIA)
- ☐ Yes
- ☐ No

Depression, Anxiety
- ☐ Yes
- ☐ No

Memory Loss, Confusion
- ☐ Yes
- ☐ No

Learning Disability such as Dyslexia, etc.
- ☐ Yes
- ☐ No

Seizures, Epilepsy
- ☐ Yes
- ☐ No

Hyperactivity, ADHD, ADD
- ☐ Yes
- ☐ No

Autism
- ☐ Yes
- ☐ No

Lymphoma or Hodgkin's disease
- ☐ Yes
- ☐ No

Other Cancer(s); please specify below
- ☐ Yes
- ☐ No
- ☐ Immediate Family Member
- ☐ Other Blood Relative

Other Condition; please specify below
- ☐ Yes
- ☐ No

If other cancer(s), birth defect(s) or other condition(s) checked above, please describe below:

Other Cancer(s): ______________________________

____

Other Condition(s): ___________________________

____

RESIDENTIAL HISTORY

1) Where do you currently live?

   Street Address: ___________________________

   City: ____________________________________

   State: ___________________________________

   Zip Code: ________________________________

   Approximately, when did you move to where you currently live?

   Date: ______________ *(mm/dd/yyyy)*

2) Is the place where you currently live located on a farm?
   - ☐ Yes
   - ☐ No
   - ☐ Don't Know

3) What best describes the geographical area where you currently live?
   - ☐ Rural, mostly farmland, agricultural fields
   - ☐ Rural, little or no farming nearby
   - ☐ Suburban, close to city, outskirts, neighborhoods
   - ☐ Urban, inner city, downtown, metropolitan area
   - ☐ Don't Know

4) Is the place where you currently live located within 1 mile of any of the following?
   - ☐ Golf Course
   - ☐ Park or Recreational Area
   - ☐ School
   - ☐ Soccer, Football, Baseball Fields
   - ☐ Cemetery
   - ☐ None of the Above

5) To the best of your knowledge, was the home where you currently live, built before 1978?
   - ☐ Yes
   - ☐ No
   - ☐ Don't Know

6) Has the home where you currently live ever been treated or sprayed for insects or rodents?
   - ☐ Yes
   - ☐ No
   - ☐ Don't Know

WATER SOURCES

1) During your pregnancy, where did your residential water come from?
   - ☐ Well
   - ☐ City, Public, or Private Water Company
   - ☐ Don't Know

   If city, public or private water selected, please list your water provider: ___________________________________

2) During your pregnancy, did you use filtered water in your home such as water faucet filtration, refrigerator water filtration, water softener, reverse osmosis, water dispenser or cooler?
   - ☐ Yes
   - ☐ No
   - ☐ Don't Know

3) During your pregnancy, what were your drinking water sources?

   ☐ Unfiltered Tap Water

   If yes, how many 8 ounces glasses of tap water do you drink per day? ________ Glasses per day

   ☐ Filtered Water

   If yes, how many 8 ounces glasses of filtered water do you drink per day? ________ Glasses per day

   ☐ Bottled Water

   If yes, how many 8 ounces glasses of bottled water do you drink per day? ________ Glasses per day

Bottled Water Chart

33.8 fluid ounces (1 liter) = 4 glasses
20 fluid ounces (0.591 liter, 591 mL) = 2 ½ glasses
16.9 fluid ounces (0.5 liter, 500 mL) = 2 glasses
12 fluid ounces (0.355 liter, 355 mL) = 1 ½ glasses
11.5 fluid ounces (0.330 liter, 330 mL) = 1 ½ glasses

SUBSTANCE USE

This information collected is for ***research purposes only*** and will not be put into your medical record or shared with your provider.

1) What best describes your use of alcohol?
   - ☐Never Used
   - ☐Currently Used
   - ☐Formerly Used

   If currently or formerly used, did you drink alcohol during your pregnancy?
   - ☐Yes
   - ☐No

   If yes, how many days per week did you drink?
   - ☐Less than 1 day per week
   - ☐Greater than or equal to 1 day per week

   If yes, on average, how many drinks did you drink per week?

   _________ Drinks per week

2) What best describes your use of tobacco?
   - ☐Never Used
   - ☐Currently Used
   - ☐Formerly Used

   If currently or formerly used, did you smoke tobacco during your pregnancy?
   - ☐Yes
   - ☐No
   - ☐Don't Know

   If yes, on average, how many did you smoke per day?

   _________ Smoked per day

3) During your pregnancy, did anyone in your household, other than yourself, smoke tobacco?
   - ☐Yes
   - ☐No
   - ☐Don't Know

4) What best describes your use of e-cigarettes or vaping?
   - ☐Never Used
   - ☐Currently Use
   - ☐Formerly Used

   If currently or formerly used, did you vape or use e-cigarettes during your pregnancy?
   - ☐Yes
   - ☐No
   - ☐Don't Know

   If yes, on average, how many times did you vape or e-cig per day?

   _________ Vapes or e-cigs per day

5) During your pregnancy, did anyone in your household, other than yourself, vape or e-cig?
   - ☐Yes
   - ☐No

6) What best describes your use of marijuana (THC), Cannabinoids, or CBD oil?
   - ☐Never Used
   - ☐Currently Use
   - ☐Formerly Used

   If currently or formerly use, did you use marijuana (THC), cannabinoids, or CBD oil during your pregnancy?
   - ☐Yes
   - ☐No
   - ☐Don't Know

7) What best describes your use of prescribed methadone, Buprenorphine, Suboxone or subutex?
   - ☐Never Used
   - ☐Currently Use
   - ☐Formerly Used

   If currently or formerly used, did you use prescribed methadone, buprenorphine, Suboxone, or subutex during your pregnancy?
   - ☐Yes
   - ☐No
   - ☐Don't Know

8) What best describes your use of other prescription drugs not prescribed to you?
   - ☐Never Used
   - ☐Currently Use
   - ☐Formerly Used

   If you currently or formerly used, did you use other prescription drugs not prescribed to you during your pregnancy?
   - ☐Yes
   - ☐No
   - ☐Don't Know

9) What best describes your use of other drugs such as heroin, cocaine, amphetamines, etc.?
   - ☐Never Used
   - ☐Currently Use
   - ☐Formerly Used

   If you currently or formerly used, did you use other drugs such as heroin, cocaine, amphetamines, etc. during your pregnancy?
   - ☐Yes
   - ☐No
   - ☐Don't Know

SPORTS, HOBBIES, TRAVEL

1) During your pregnancy, did you do any of the following sports, hobbies, crafts, recreational activities?

Water sports such as swimming, skiing, tubing, surfing, etc.
- ☐ Never/Rarely
- ☐ Daily
- ☐ Weekly
- ☐ Monthly

Underwater sports such as snorkeling, scuba diving, etc.
- ☐ Never/Rarely
- ☐ Daily
- ☐ Weekly
- ☐ Monthly

Boating, sailing, canoeing, rafting, kayaking, etc.
- ☐ Never/Rarely
- ☐ Daily
- ☐ Weekly
- ☐ Monthly

Camping, hiking, fishing, hunting, etc.
- ☐ Never/Rarely
- ☐ Daily
- ☐ Weekly
- ☐ Monthly

Biking, running, jogging, walking, etc.
- ☐ Never/Rarely
- ☐ Daily
- ☐ Weekly
- ☐ Monthly

Golfing, driving range
- ☐ Never/Rarely
- ☐ Daily
- ☐ Weekly
- ☐ Monthly

Rock or mountain climbing, sky or cliff diving, parasailing
- ☐ Never/Rarely
- ☐ Daily
- ☐ Weekly
- ☐ Monthly

Winter sports such as skiing, snowboarding, snowmobiling
- ☐ Never/Rarely
- ☐ Daily
- ☐ Weekly
- ☐ Monthly

Attended/played outdoor field sports (soccer, football, etc.)
- ☐ Never/Rarely
- ☐ Daily
- ☐ Weekly
- ☐ Monthly

Sitting in saunas or hot tubs
- ☐ Never/Rarely
- ☐ Daily
- ☐ Weekly
- ☐ Monthly

Jewelry/pottery making, stained glass, oil painting, ceramics
- ☐ Never/Rarely
- ☐ Daily
- ☐ Weekly
- ☐ Monthly

Other, please specify: ____________________________
- ☐ Never/Rarely
- ☐ Daily
- ☐ Weekly
- ☐ Monthly

2) During your pregnancy, did you travel outside of the U.S.?
- ☐ Yes
- ☐ No
- ☐ Don't Know

If yes, how many times did you travel outside of the U.S.?

_________ Times

If yes, what country(s) did you visit?

____

If yes, approximately how many weeks were you outside of the U.S.? _________ Weeks

If yes, were you outside of the U.S. on any one trip for greater than 1 week?
- ☐ Yes
- ☐ No
- ☐ Don't Know

If yes, were any of the places that you visited near agriculture fields or farmland?
- ☐ Yes
- ☐ No
- ☐ Don't Know

HOME RELATED ACTIVITIES

Instructions: Please check how frequency you used conventional chemicals (products that contain chemicals) and eco-friendly (products that contain natural ingredients; also referred to as biochemical, biodegradable, environmental friendly or green products).

1) During your pregnancy, how many times did you handle, mix, or apply chemicals or fertilizers while gardening, farming, landscaping, lawn care, or other activities?

To manage or kill insects:

Conventional	***Eco-Friendly***
☐ Never/Rarely	☐ Never/Rarely
☐ 1-2 times	☐ 1-2 times
☐ 3-5 times	☐ 3-5 times
☐ 5 times or more	☐ 5 times or more

To manage or kill weeds:

Conventional	***Eco-Friendly***
☐ Never/Rarely	☐ Never/Rarely
☐ 1-2 times	☐ 1-2 times
☐ 3-5 times	☐ 3-5 times
☐ 5 times or more	☐ 5 times or more

To control plant molds or fungal diseases:

Conventional	***Eco-Friendly***
☐ Never/Rarely	☐ Never/Rarely
☐ 1-2 times	☐ 1-2 times
☐ 3-5 times	☐ 3-5 times
☐ 5 times or more	☐ 5 times or more

To fertilize your lawns, gardens, etc.:

Conventional	***Eco-Friendly***
☐ Never/Rarely	☐ Never/Rarely
☐ 1-2 times	☐ 1-2 times
☐ 3-5 times	☐ 3-5 times
☐ 5 times or more	☐ 5 times or more

2) If known, please list the chemicals you referred to in your responses above:

Used to manage or kill insects:

____

Used to manager or kill weeds:

____

Used to control plant molds or fungal diseases:

____

Used to fertilize your lawns, gardens, etc.:

____

HOUSEHOLD CLEANING INFORMATION

1) During your pregnancy, how frequently did you use the following household cleaners?

Sanitizers, disinfectants (e.g. Lysol, Clorox, etc.):

Conventional	***Eco-Friendly***
☐Never/Rarely	☐Never/Rarely
☐Daily	☐Daily
☐Weekly	☐Weekly
☐Monthly	☐Monthly

Chlorine Bleach:

Conventional	***Eco-Friendly***
☐Never/Rarely	☐Never/Rarely
☐Daily	☐Daily
☐Weekly	☐Weekly
☐Monthly	☐Monthly

Detergents or Stain Removers:

Conventional	***Eco-Friendly***
☐Never/Rarely	☐Never/Rarely
☐Daily	☐Daily
☐Weekly	☐Weekly
☐Monthly	☐Monthly

Carpet or Upholstery Cleaners

Conventional	***Eco-Friendly***
☐Never/Rarely	☐Never/Rarely
☐Daily	☐Daily
☐Weekly	☐Weekly
☐Monthly	☐Monthly

Furniture or Floor Polish

Conventional	***Eco-Friendly***
☐Never/Rarely	☐Never/Rarely
☐Daily	☐Daily
☐Weekly	☐Weekly
☐Monthly	☐Monthly

Oven Cleaners

Conventional	***Eco-Friendly***
☐Never/Rarely	☐Never/Rarely
☐Daily	☐Daily
☐Weekly	☐Weekly
☐Monthly	☐Monthly

Air or Carpet Fresheners/Deodorizers

Conventional	***Eco-Friendly***
☐Never/Rarely	☐Never/Rarely
☐Daily	☐Daily
☐Weekly	☐Weekly
☐Monthly	☐Monthly

Dry Cleaning Chemicals:

Conventional	***Eco-Friendly***
☐Never/Rarely	☐Never/Rarely
☐Daily	☐Daily
☐Weekly	☐Weekly
☐Monthly	☐Monthly

2) During your pregnancy, did you clean homes, launder clothes or provide professional cleaning services other than for your own household?
   - ☐ Yes
   - ☐ No

If yes, how frequently did you *clean homes*?
- ☐ Never/Rarely
- ☐ Daily
- ☐ Weekly
- ☐ Monthly

If yes, how frequently did you *launder clothes*?
- ☐ Never/Rarely
- ☐ Daily
- ☐ Weekly
- ☐ Monthly

If yes, how frequently did you *provide professional cleaning services*?
- ☐ Never/Rarely
- ☐ Daily
- ☐ Weekly
- ☐ Monthly

OCCUPATION AND WORK ENVIRONMENT

1) During your pregnancy, what was your work situation/status? *Check all that apply*
   - ☐ Employed
   - ☐ Intern/Apprentice
   - ☐ Unemployed/Laid Off
   - ☐ Stay at home (do not work)
   - ☐ Rehabilitation/Disabled
   - ☐ Other, please describe below:

   ____

2) During your pregnancy, did you work outside your home?
   - ☐ Yes
   - ☐ No

   If yes, how many hours per week did you work outside your home?

   ________ Hours per week

   If yes, was your workplace located on or close to a farm?
   - ☐ Yes
   - ☐ No
   - ☐ Don't Know

   If yes, describe the geographical area where your workplace was located:
   - ☐ Rural, mostly farmland, agricultural fields
   - ☐ Rural, little or no farming nearby
   - ☐ Suburban, close to city, outskirts, neighborhoods
   - ☐ Urban, inner city, downtown, metropolitan area
   - ☐ Don't Know

   If yes, was your workplace located within 1 mile of any of the following?
   - ☐ Golf Course
   - ☐ Park or Recreational Area
   - ☐ School
   - ☐ Soccer, Football, Baseball Fields
   - ☐ Cemetery

   If yes, please describe the type of work you carried out as accurately as possible (e.g. hospital department for children with cancer, body shop at a garage for diesel vehicles, farming with dairy cows, etc.):

   ____

   ____

3) During your pregnancy, did you mix, handle, or apply any of the following chemicals for work?

   Petroleum plastics (e.g. propylene or butylene glycol, etc.)
   - ☐ Yes
   - ☐ No
   - ☐ Don't Know

   Chrome, arsenic, cadmium, lead, mercury
   - ☐ Yes
   - ☐ No
   - ☐ Don't Know

   Gasoline or exhaust other than when filling gasoline in your car
   - ☐ Yes
   - ☐ No
   - ☐ Don't Know

   Pesticides such as weed killer, insecticides, fungicides
   - ☐ Yes
   - ☐ No
   - ☐ Don't Know

   Laughing gas or other anesthetic gases other than as a patient
   - ☐ Yes
   - ☐ No
   - ☐ Don't Know

   Mouse or rat poisons
   - ☐ Yes
   - ☐ No
   - ☐ Don't Know

   Chemotherapeutic substances/treatment other than as a patient
   - ☐ Yes
   - ☐ No
   - ☐ Don't Know

   Oil /water-based latex paints, paint thinners, lacquer, glue remover
   - ☐ Yes
   - ☐ No
   - ☐ Don't Know

   Industrial dyes/ink, photographic chemicals, fixatives, developers
   - ☐ Yes
   - ☐ No
   - ☐ Don't Know

   Motor oil, lubrication oil or other types of oils
   - ☐ Yes
   - ☐ No
   - ☐ Don't Know

OCCUPATION AND WORK ENVIRONMENT continued

Welding, soldering, etc.
- ☐ Yes
- ☐ No
- ☐ Don't Know

Formalin/formaldehyde
- ☐ Yes
- ☐ No
- ☐ Don't Know

Dry cleaning fluids (i.e. PERC, etc.)
- ☐ Yes
- ☐ No
- ☐ Don't Know

Foam, fiberglass, blown-in insulation, etc.
- ☐ Yes
- ☐ No
- ☐ Don't Know

Other, please specify: ______________________________
- ☐ Yes
- ☐ No
- ☐ Don't Know

MILITARY

1) At any time, have you ever served in the military including Reserve or National Guard?
   - ☐ Yes
   - ☐ No
   - ☐ Don't Know

   If yes, in what countries were you stationed?

   ____

   ____

   If yes, what is your current status?
   - ☐ Active Duty
   - ☐ Reserve
   - ☐ National Guard
   - ☐ Veteran

If yes, to the best of your knowledge, were you exposed to any of the following chemicals?
- ☐ Agent Orange or other herbicides used to destroy foliage/crops
- ☐ Pesticides used to repel or destroy insects and pathogens
- ☐ Industrial Chemicals found on some military bases
- ☐ PCBs typically used as coolant and insulating fluid
- ☐ Depleted Uranium used in military tank armor and bullets
- ☐ PFAs typically found in products including fire-fighting foams
- ☐ Shrapnel/toxic metals that remained in your body after injury
- ☐ Sand, Dust, and Particulates
- ☐ Sulfur Fire
- ☐ Ionizing Radiation, X Rays, Nuclear fallout
- ☐ Chromium
- ☐ Burn Pits used to dispose of military site waste
- ☐ None that I'm aware of
- ☐ Other, please specify below:

  ____

  ____

COVID-19

1) During your pregnancy, did you suspect that you or someone in your household had COVID-19?
   - ☐ Yes
   - ☐ No
   - ☐ Possibly
   - ☐ Don't Know

2) During your pregnancy, did you have any symptoms (e.g. fever, dry cough, sore throat, difficulty breathing or shortness of breath, chest pain or pressure, fatigue, body or muscle aches, loss of appetite, loss of taste or smell, headache, chills or repeated shaking, nausea or vomiting, diarrhea or stomach problems)?
   - ☐ Yes
   - ☐ No
   - ☐ Unsure

   If yes, did you take medications to treat your symptoms?
   - ☐ Yes
   - ☐ No

   If yes, what medications did you take?

   ____

   ____

3) During your pregnancy, has anyone in your household other than yourself tested positive for COVID-19?
   - ☐ Yes
   - ☐ No
   - ☐ Unsure

4) During your pregnancy, were you ever tested for COVID-19?
   - ☐ Yes
   - ☐ No

   If yes, did you ever test positive for COVID-19?
   - ☐ Yes
   - ☐ No

   If yes, when did you test positive for COVID-19?

   Date _______________ (mm/dd/yyyy)

   Date _______________ (mm/dd/yyyy)

5) Did you self-quarantine due to COVID-19?
   - ☐ Yes
   - ☐ No

   If yes, how many days were you in self-quarantine?

   _________ Days

6) Were you ever seen by a doctor or an ER visit due to COVID-19 (confirmed or suspected)?
   - ☐ Yes
   - ☐ No

7) Were you admitted to the hospital for treatment?
   - ☐ Yes
   - ☐ No

   If yes, approximately when were you in the hospital?

   Date admitted ________________ (mm/dd/yyyy)

   Date discharged _______________ (mm/dd/yyyy)

   If yes, were you put on a ventilator to help you breathe?
   - ☐ Yes
   - ☐ No

   If yes, number of days on ventilator?

   _________ Days

   If yes, once released from the hospital did you self-quarantine?
   - ☐ Yes
   - ☐ No

   If yes, number of days in self-quarantine?

   ________ Days

IRB: 1906366316

Subject ID#:_____________

Circle Visit: 0-13 Wk or 14-27 Wk or 28-42 wk or Postpartum

PSS

INSTRUCTIONS:

The questions in this scale ask you about your feelings and thoughts during THE LAST MONTH. In each case, please indicate your response by placing an "X" over the circle representing HOW OFTEN you felt or thought a certain way.

	Never<br>0	Almost Never<br>1	Sometimes<br>2	Fairly Often<br>3	Very Often<br>4
**1. In the last month, how often have you been upset because of something that happened unexpectedly?**	○	○	○	○	○
**2. In the last month, how often have you felt that you were unable to control the important things in your life?**	○	○	○	○	○
**3. In the last month, how often have you felt nervous and "stressed"?**	○	○	○	○	○
**4. In the last month, how often have you felt confident about your ability to handle your personal problems?**	○	○	○	○	○
**5. In the last month, how often have you felt that things were going your way?**	○	○	○	○	○
**6. In the last month, how often have you found that you could not cope with all the things that you had to do?**	○	○	○	○	○
**7. In the last month, how often have you been able to control irritations in your life?**	○	○	○	○	○
**8. In the last month, how often have you felt that you were on top of things?**	○	○	○	○	○
**9. In the last month, how often have you been angered because of things that were outside your control?**	○	○	○	○	○
**10. In the last month, how often have you felt difficulties were piling up so high that you could not overcome them?**	○	○	○	○	○

For Office Use Only:

Initials of Person Entering Form:____________

Date Form Entered:_____________

Resize font:

Food Frequency Questionnaire

Please complete the survey below.

Thank you!

FRESH FRUITS - Tree Fruits

FRESH FRUITS - Tree Fruits

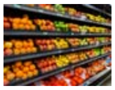

Which of the FRESH FRUITS listed below did you eat during the three months prior to conception? Check all that apply.

- [x] Apples
- [x] Oranges
- [x] Peaches
- [x] Pears
- [x] Plums
- [x] Grapefruit
- [x] Nectarines
- [x] Mangoes
- [x] Kiwi Fruits
- [x] Papayas
- [x] Bananas
- [ ] None

Fresh Apples

Serving Size: 1 medium apple (size of a tennis ball) or 1 cup, sliced or chopped, raw or cooked

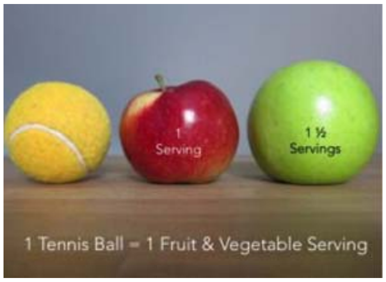

During the three months prior to conception, on average, how many servings of fresh apples did you consume per week.

- ○ 1 or less apples per week
- ○ 2 to 4 apples per week
- ○ 5 to 7 apples per week
- ○ 8 to 10 apples per week
- ○ 11 to 13 apples per week
- ○ 14 or more apples per week

reset

Protocol 1906366316 IRB Approved

Please indicate what percentage of the fresh apples that you consumed were organic.

- 0% to 10%
- 11% to 33%
- 33% to 66%
- 67% to 90%
- 90% to 100%

reset

Fresh Oranges

Serving Size: 1 large orange (size of a baseball) or 1 cup of orange wedges

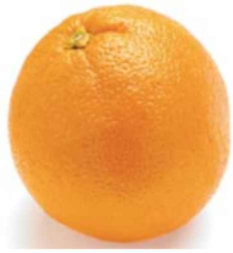

During the three months prior to conception, on average, how many servings of fresh oranges did you consume per week.

- 1 or less oranges per week
- 2 to 4 oranges per week
- 5 to 7 oranges per week
- 8 to 10 oranges per week
- 11 to 13 oranges per week
- 14 or more oranges per week

reset

Please indicate what percentage of the fresh oranges that you consumed were organic.

- 0% to 10%
- 11% to 33%
- 33% to 66%
- 67% to 90%
- 90% to 100%

reset

Fresh Peaches

Serving Size: 1 large peach (2 3/4" diameter) or 1 cup of sliced or diced

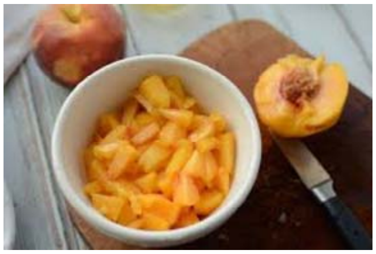

Protocol 1906366316 IRB Approved

During the three months prior to conception, on average, how many servings of fresh peaches did you consume per week.

- 1 or less peaches per week
- 2 to 4 peaches per week
- 5 to 7 peaches per week
- 8 to 10 peaches per week
- 11 to 13 peaches per week
- 14 or more peaches per week

reset

Please indicate what percentage of the fresh peaches that you consumed were organic.

- 0% to 10%
- 11% to 33%
- 33% to 66%
- 67% to 90%
- 90% to 100%

reset

Fresh Pears

Serving Size: 1 medium pear or 1 cup sliced or diced

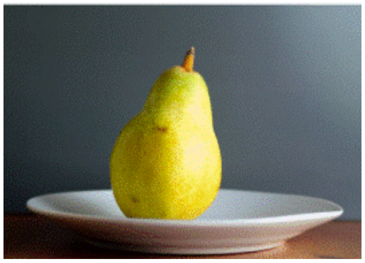

During the three months prior to conception, on average, how many servings of fresh pears did you consume per week.

- 1 or less pears per week
- 2 to 4 pears per week
- 5 to 7 pears per week
- 8 to 10 pears per week
- 11 to 13 pears per week
- 14 or more pears per week

reset

Please indicate what percentage of the fresh pears that you consumed were organic.

- 0% to 10%
- 11% to 33%
- 33% to 66%
- 67% to 90%
- 90% to 100%

reset

Protocol 1906366316 IRB Approved

Fresh Plums

Serving Size: 2 large plums (size of a tennis ball)

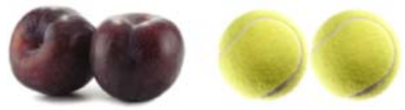

**During the three months prior to conception, on average,
how many servings of fresh plums did you consume per week.**

- ◯ 1 or less plums per week
- ◯ 2 to 4 plums per week
- ◯ 5 to 7 plums per week
- ◯ 8 to 10 plums per week
- ◯ 11 to 13 plums per week
- ◯ 14 or more plums per week

reset

Please indicate what percentage of the fresh plums that you consumed were organic.

- ◯ 0% to 10%
- ◯ 11% to 33%
- ◯ 33% to 66%
- ◯ 67% to 90%
- ◯ 90% to 100%

reset

Fresh Grapefruit

Serving Size: 1 medium grapefruit (size of a softball)

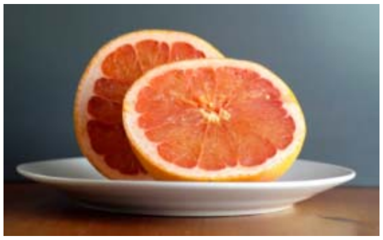

**During the three months prior to conception, on average,
how many servings of fresh grapefruits did you consume per week.**

- ◯ 1 or less servings per week
- ◯ 2 to 4 servings per week
- ◯ 5 to 7 servings per week
- ◯ 8 to 10 servings per week
- ◯ 11 to 13 servings per week
- ◯ 14 or more servings per week

reset

Protocol 1906366316 IRB Approved

Please indicate what percentage of the fresh grapefruits that you consumed were organic.

- 0% to 10%
- 11% to 33%
- 33% to 66%
- 67% to 90%
- 90% to 100%

reset

Fresh Nectarines

Serving Size: 1 large nectarine (2 3/4" diameter) or 1 cup of sliced or diced

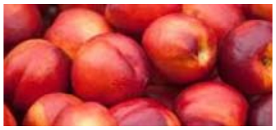

During the three months prior to conception, on average, how many servings of fresh nectarines did you consume per week.

- 1 or less nectarines per week
- 2 to 4 nectarines per week
- 5 to 7 nectarines per week
- 8 to 10 nectarines per week
- 11 to 13 nectarines per week
- 14 or more nectarines per week

reset

Please indicate what percentage of the fresh nectarines that you consumed were organic.

- 0% to 10%
- 11% to 33%
- 33% to 66%
- 67% to 90%
- 90% to 100%

reset

Fresh Mangoes

Serving Size: 3/4 cup of mango

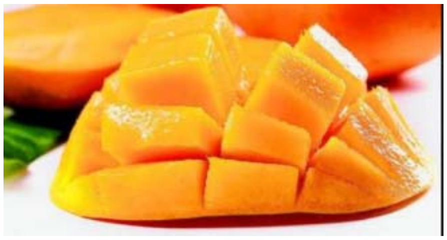

Protocol 1906366316 IRB Approved

During the three months prior to conception, on average, how many servings of fresh mangoes did you consume per week.

- 1 or less mangoes per week
- 2 to 4 mangoes per week
- 5 to 7 mangoes per week
- 8 to 10 mangoes per week
- 11 to 13 mangoes per week
- 14 or more mangoes per week

reset

Please indicate what percentage of the fresh mangoes that you consumed were organic.

- 0% to 10%
- 11% to 33%
- 33% to 66%
- 67% to 90%
- 90% to 100%

reset

Fresh Kiwi Fruit

Serving Size: 2 small kiwis

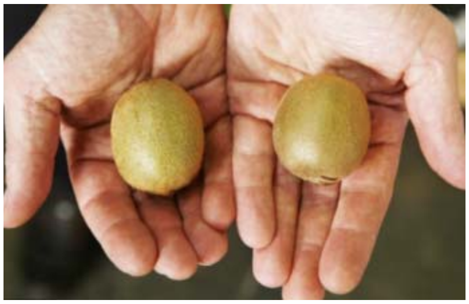

During the three months prior to conception, on average, how many servings of fresh kiwi fruit did you consume per week.

- 1 or less kiwi fruits per week
- 2 to 4 kiwi fruits per week
- 5 to 7 kiwi fruits per week
- 8 to 10 kiwi fruits per week
- 11 to 13 kiwi fruits per week
- 14 or more kiwi fruits per week

reset

Please indicate what percentage of the fresh kiwi fruits you consumed were organic.

- 0% to 10%
- 11% to 33%
- 33% to 66%
- 67% to 90%
- 90% to 100%

reset

Protocol 1906366316 IRB Approved

Fresh Papayas

Serving Size: 1 slice of papaya approx. 1.5 inches thick

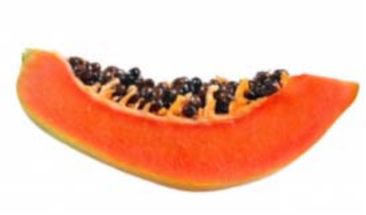

During the three months prior to conception, on average, how many servings of fresh papayas did you consume per week.

- 1 or less papayas per week
- 2 to 4 papayas per week
- 5 to 7 papayas per week
- 8 to 10 papayas per week
- 11 to 13 papayas per week
- 14 or more papayas per week

reset

Please indicate what percentage of the fresh papayas you consumed were organic.

- 0% to 10%
- 11% to 33%
- 33% to 66%
- 67% to 90%
- 90% to 100%

reset

Fresh Bananas

Serving Size: 1 large (8"-9" long) or 1 cup sliced

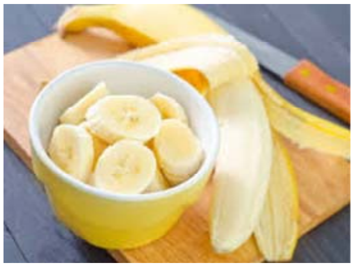

During the three months prior to conception, on average, how many servings of fresh bananas did you consume per week.

- 1 or less bananas per week
- 2 to 4 bananas per week
- 5 to 7 bananas per week
- 8 to 10 bananas per week
- 11 to 13 bananas per week
- 14 or more bananas per week

reset

Protocol 1906366316 IRB Approved

Please indicate what percentage of the fresh bananas you consumed were organic.

- ○ 0% to 10%
- ○ 11% to 33%
- ○ 33% to 66%
- ○ 67% to 90%
- ○ 90% to 100%

reset

FRESH FRUITS - Berries

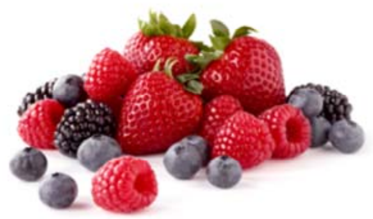

Which of the FRESH FRUITS listed below did you eat during the three months prior to conception? Check all that apply.

- ☑ Strawberries
- ☑ Blueberries
- ☑ Raspberries
- ☑ Blackberries
- ☑ Cherries
- ☑ Grapes
- ☑ Cranberries
- ☐ None

Fresh Strawberries

Serving Size: 8 strawberries (1 cup)

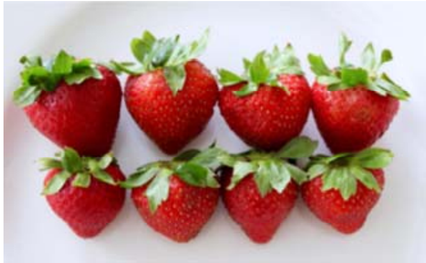

During the three months prior to conception, on average, how many servings of fresh strawberries did you consume per week.

- ○ 1 or less servings per week
- ○ 2 to 4 servings per week
- ○ 5 to 7 servings per week
- ○ 8 to 10 servings per week
- ○ 11 to 13 servings per week
- ○ 14 or more servings per week

reset

Protocol 1906366316 IRB Approved

Please indicate what percentage of the fresh strawberries you consumed were organic.

- ◯ 0% to 10%
- ◯ 11% to 33%
- ◯ 33% to 66%
- ◯ 67% to 90%
- ◯ 90% to 100%

reset

Fresh Blueberries

Serving Size: 78 blueberres (1 cup)

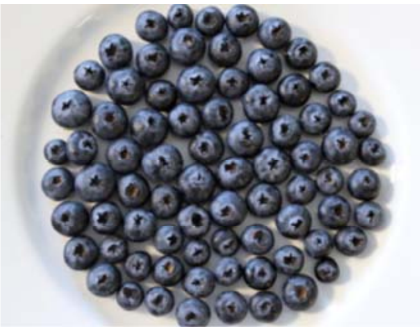

During the three months prior to conception, on average, how many servings of fresh blueberries did you consume per week.

- ◯ 1 or less servings per week
- ◯ 2 to 4 servings per week
- ◯ 5 to 7 servings per week
- ◯ 8 to 10 servings per week
- ◯ 11 to 13 servings per week
- ◯ 14 or more servings per week

reset

Please indicate what percentage of the fresh blueberries you consumed were organic.

- ◯ 0% to 10%
- ◯ 11% to 33%
- ◯ 33% to 66%
- ◯ 67% to 90%
- ◯ 90% to 100%

reset

Fresh Raspberries

Serving Size: 30 Raspberries (1 cup)

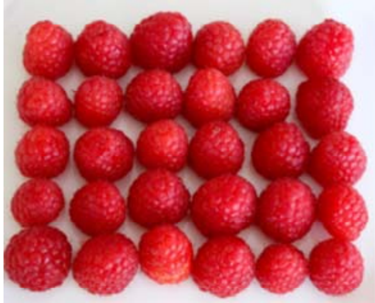

Protocol 1906366316 IRB Approved

During the three months prior to conception, on average, how many servings of fresh raspberries did you consume per week.

- ◯ 1 or less servings per week
- ◯ 2 to 4 servings per week
- ◯ 5 to 7 servings per week
- ◯ 8 to 10 servings per week
- ◯ 11 to 13 servings per week
- ◯ 14 or more servings per week

reset

Please indicate what percentage of the fresh raspberries you consumed were organic.

- ◯ 0% to 10%
- ◯ 11% to 33%
- ◯ 33% to 66%
- ◯ 67% to 90%
- ◯ 90% to 100%

reset

Fresh Blackberries

Serving Size: 1 cup or 20 blackberries

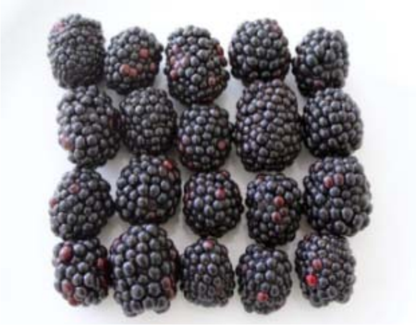

During the three months prior to conception, on average, how many servings of fresh blackberries did you consume per week.

- ◯ 1 or less servings per week
- ◯ 2 to 4 servings per week
- ◯ 5 to 7 servings per week
- ◯ 8 to 10 servings per week
- ◯ 11 to 13 servings per week
- ◯ 14 or more servings per week

reset

Please indicate what percentage of the fresh blackberries you consumed were organic.

- ◯ 0% to 10%
- ◯ 11% to 33%
- ◯ 33% to 66%
- ◯ 67% to 90%
- ◯ 90% to 100%

reset

Protocol 1906366316 IRB Approved

Fresh Cherries

Serving Size: 1 cup or 20 cherries

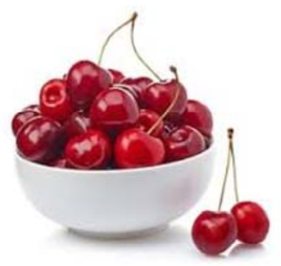

During the three months prior to conception, on average, how many servings of fresh cherries did you consume per week.

- ◯ 1 or less servings per week
- ◯ 2 to 4 servings per week
- ◯ 5 to 7 servings per week
- ◯ 8 to 10 servings per week
- ◯ 11 to 13 servings per week
- ◯ 14 or more servings per week

reset

Please indicate what percentage of the fresh cherries you consumed were organic.

- ◯ 0% to 10%
- ◯ 11% to 33%
- ◯ 33% to 66%
- ◯ 67% to 90%
- ◯ 90% to 100%

reset

Fresh Grapes

Serving Size: 1 cup or 36 grapes

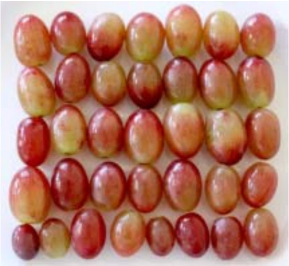

During the three months prior to conception, on average, how many servings of fresh grapes did you consume per week.

- ◯ 1 or less servings per week
- ◯ 2 to 4 servings per week
- ◯ 5 to 7 servings per week
- ◯ 8 to 10 servings per week
- ◯ 11 to 13 servings per week
- ◯ 14 or more servings per week

reset

Protocol 1906366316 IRB Approved

Please indicate what percentage of the fresh grapes you consumed were organic.

- ◯ 0% to 10%
- ◯ 11% to 33%
- ◯ 33% to 66%
- ◯ 67% to 90%
- ◯ 90% to 100%

reset

Fresh Cranberries

Serving Size: 1 cup

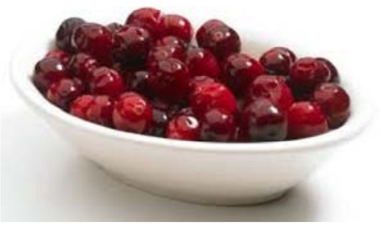

During the three months prior to conception, on average,
how many servings of fresh cranberries did you consume per week.

- ◯ 1 or less servings per week
- ◯ 2 to 4 servings per week
- ◯ 5 to 7 servings per week
- ◯ 8 to 10 servings per week
- ◯ 11 to 13 servings per week
- ◯ 14 or more servings per week

reset

Please indicate what percentage of the fresh cranberries you consumed were organic.

- ◯ 0% to 10%
- ◯ 11% to 33%
- ◯ 33% to 66%
- ◯ 67% to 90%
- ◯ 90% to 100%

reset

FRESH FRUITS - Melons,Cantaloupes & Pineapple

Melons, Cantaloupes & Pineapple

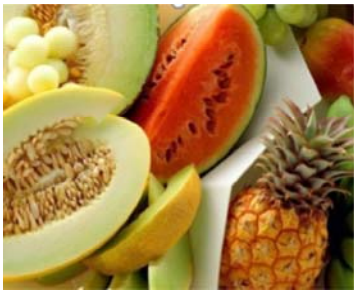

Protocol 1906366316 IRB Approved

Which of the FRESH FRUITS listed below did you eat during the <u>three months prior to conception?</u> Check all that apply.

- [x] Watermelons
- [x] Cantaloupes
- [x] Pineapples
- [ ] None

Fresh Watermelons

Serving Size: 1 small (1" thick) wedge, or 1 cup, diced or 6 melon balls

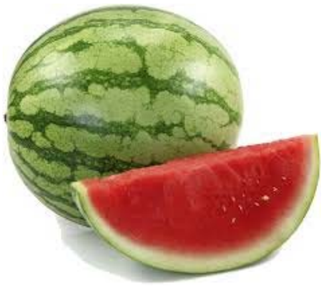

During the three months prior to conception, on average, how many servings of fresh watermelon did you consume per week.

- ○ 1 or less servings per week
- ○ 2 to 4 servings per week
- ○ 5 to 7 servings per week
- ○ 8 to 10 servings per week
- ○ 11 to 13 servings per week
- ○ 14 or more servings per week

reset

Please indicate what percentage of the fresh watermelon you consumed were organic.

- ○ 0% to 10%
- ○ 11% to 33%
- ○ 33% to 66%
- ○ 67% to 90%
- ○ 90% to 100%

reset

Fresh Cantaloupes

Serving Size:1 cup cubed or two slices

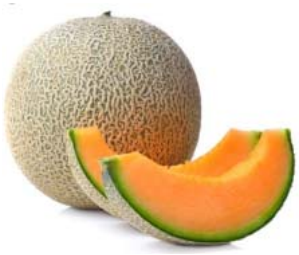

Protocol 1906366316 IRB Approved

During the three months prior to conception, on average, how many servings of fresh cantaloupe did you consume per week.

- 1 or less servings per week
- 2 to 4 servings per week
- 5 to 7 servings per week
- 8 to 10 servings per week
- 11 to 13 servings per week
- 14 or more servings per week

reset

Please indicate what percentage of the fresh cantaloupe you consumed were organic.

- 0% to 10%
- 11% to 33%
- 33% to 66%
- 67% to 90%
- 90% to 100%

reset

Fresh Pineapples

Serving Size of Pineapple: 1 cup

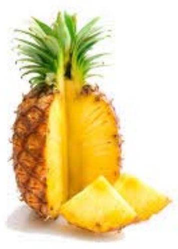

During the three months prior to conception, on average, how many servings of fresh pineapple did you consume per week.

- 1 or less servings per week
- 2 to 4 servings per week
- 5 to 7 servings per week
- 8 to 10 servings per week
- 11 to 13 servings per week
- 14 or more servings per week

reset

Please indicate what percentage of the fresh pineapple you consumed were organic.

- 0% to 10%
- 11% to 33%
- 33% to 66%
- 67% to 90%
- 90% to 100%

reset

FRESH VEGETABLES - Leafy Greens & Edible Stems

Protocol 1906366316 IRB Approved

Leafy Greens & Edible Stems

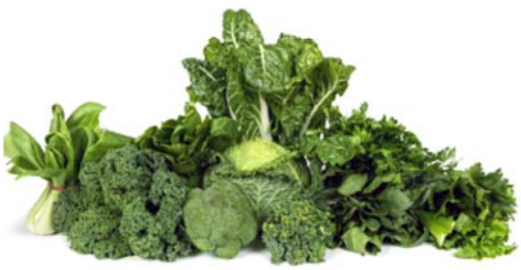

Which of the FRESH VEGETABLES listed below did you eat during the three months prior to conception? Check all that apply.

- [x] Lettuce
- [x] Spinach
- [x] Kale
- [x] Cabbage
- [x] Collard Greens
- [x] Broccoli
- [x] Cauliflower
- [x] Asparagus
- [x] Celery
- [ ] None

Fresh Lettuce

Serving Size: 2 cups of shredded lettuce

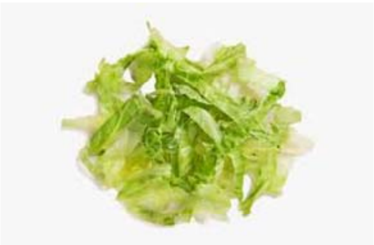

During the three months prior to conception, on average, how many servings of fresh lettuce did you consume per week.

- ○ 1 or less servings per week
- ○ 2 to 4 servings per week
- ○ 5 to 7 servings per week
- ○ 8 to 10 servings per week
- ○ 11 to 13 servings per week
- ○ 14 or more servings per week

reset

Please indicate what percentage of the fresh lettuce you consumed were organic.

- ○ 0% to 10%
- ○ 11% to 33%
- ○ 33% to 66%
- ○ 67% to 90%
- ○ 90% to 100%

reset

Protocol 1906366316 IRB Approved

Fresh Spinach

Serving Size of Spinach: 1 cup cooked or 2 cups raw

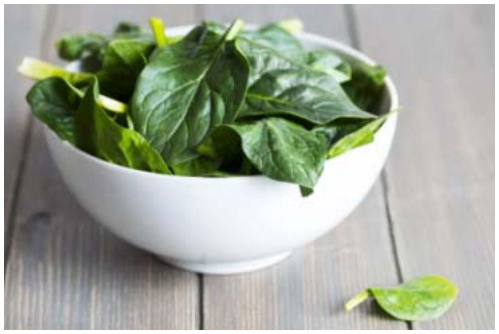

During the three months prior to conception, on average, how many servings of fresh spinach did you consume per week.

- ◯ 1 or less servings per week
- ◯ 2 to 4 servings per week
- ◯ 5 to 7 servings per week
- ◯ 8 to 10 servings per week
- ◯ 11 to 13 servings per week
- ◯ 14 or more servings per week

reset

Please indicate what percentage of the fresh spinach you consumed were organic.

- ◯ 0% to 10%
- ◯ 11% to 33%
- ◯ 33% to 66%
- ◯ 67% to 90%
- ◯ 90% to 100%

reset

Fresh Kale

Serving Size of Kale: 2 cups raw (medium bowl)

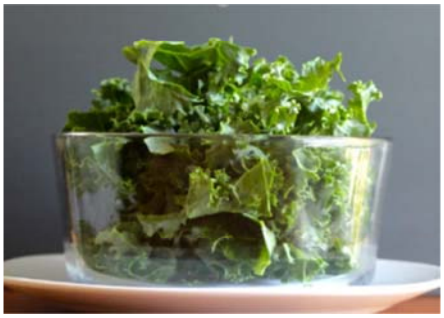

Protocol 1906366316 IRB Approved

During the three months prior to conception, on average, how many servings of fresh kale did you consume per week.

- ○ 1 or less servings per week
- ○ 2 to 4 servings per week
- ○ 5 to 7 servings per week
- ○ 8 to 10 servings per week
- ○ 11 to 13 servings per week
- ○ 14 or more servings per week

reset

Please indicate what percentage of the fresh kale you consumed were organic.

- ○ 0% to 10%
- ○ 11% to 33%
- ○ 33% to 66%
- ○ 67% to 90%
- ○ 90% to 100%

reset

Fresh Cabbage

Serving Size of Cabbage: 1 cup - wedge, chopped or shredded

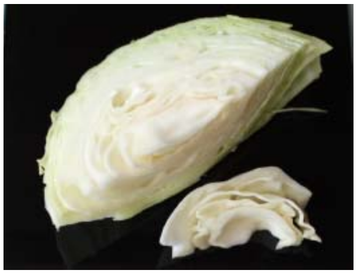

During the three months prior to conception, on average, how many servings of fresh cabbage did you consume per week.

- ○ 1 or less servings per week
- ○ 2 to 4 servings per week
- ○ 5 to 7 servings per week
- ○ 8 to 10 servings per week
- ○ 11 to 13 servings per week
- ○ 14 or more servings per week

reset

Please indicate what percentage of the fresh cabbage you consumed were organic.

- ○ 0% to 10%
- ○ 11% to 33%
- ○ 33% to 66%
- ○ 67% to 90%
- ○ 90% to 100%

reset

Protocol 1906366316 IRB Approved

Fresh Collard Greens

Serving Size of Collard Greens: 2 cups chopped or medium bowl

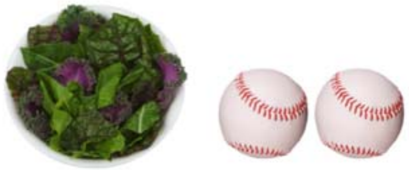

During the three months prior to conception, on average, how many servings of fresh collard greens did you consume per week.

- 1 or less servings per week
- 2 to 4 servings per week
- 5 to 7 servings per week
- 8 to 10 servings per week
- 11 to 13 servings per week
- 14 or more servings per week

reset

Please indicate what percentage of the fresh collard greens you consumed were organic.

- 0% to 10%
- 11% to 33%
- 33% to 66%
- 67% to 90%
- 90% to 100%

reset

Fresh Broccoli

Serving Size of Broccoli: 1 cup or six pieces

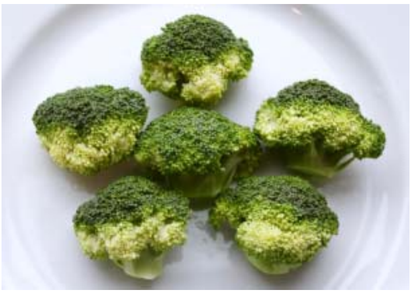

During the three months prior to conception, on average, how many servings of fresh broccoli did you consume per week.

- 1 or less servings per week
- 2 to 4 servings per week
- 5 to 7 servings per week
- 8 to 10 servings per week
- 11 to 13 servings per week
- 14 or more servings per week

reset

Protocol 1906366316 IRB Approved

Please indicate what <u>percentage</u> of the fresh broccoli you consumed were <u>organic</u>.

- ◯ 0% to 10%
- ◯ 11% to 33%
- ◯ 33% to 66%
- ◯ 67% to 90%
- ◯ 90% to 100%

reset

Fresh Cauliflower

Serving Size of Cauliflower: 1 cup of florets or cauliflower rice

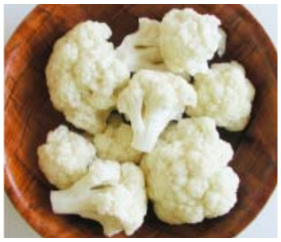

During the three months prior to conception, on average, how many servings of fresh cauliflower did you <u>consume per week</u>.

- ◯ 1 or less servings per week
- ◯ 2 to 4 servings per week
- ◯ 5 to 7 servings per week
- ◯ 8 to 10 servings per week
- ◯ 11 to 13 servings per week
- ◯ 14 or more servings per week

reset

Please indicate what <u>percentage</u> of the fresh cauliflower you consumed were <u>organic</u>.

- ◯ 0% to 10%
- ◯ 11% to 33%
- ◯ 33% to 66%
- ◯ 67% to 90%
- ◯ 90% to 100%

reset

Fresh Asparagus

Serving Size of Asparagus: 4 large or 6 medium spears

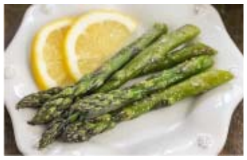

Protocol 1906366316 IRB Approved

During the three months prior to conception, on average, how many servings of fresh asparagus did you consume per week.

- 1 or less servings per week
- 2 to 4 servings per week
- 5 to 7 servings per week
- 8 to 10 servings per week
- 11 to 13 servings per week
- 14 or more servings per week

reset

Please indicate what percentage of the fresh asparagus you consumed were organic.

- 0% to 10%
- 11% to 33%
- 33% to 66%
- 67% to 90%
- 90% to 100%

reset

Fresh Celery

Serving Size of Celery: 2 large stocks or 1 cup diced

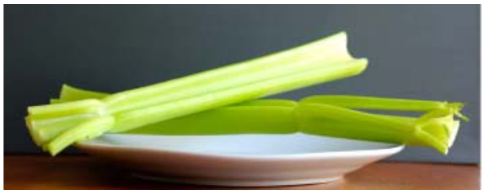

During the three months prior to conception, on average, how many servings of fresh celery did you consume per week.

- 1 or less servings per week
- 2 to 4 servings per week
- 5 to 7 servings per week
- 8 to 10 servings per week
- 11 to 13 servings per week
- 14 or more servings per week

reset

Please indicate what percentage of the fresh celery you consumed were organic.

- 0% to 10%
- 11% to 33%
- 33% to 66%
- 67% to 90%
- 90% to 100%

reset

FRESH VEGETABLES - Fruiting Vegetables

Protocol 1906366316 IRB Approved

Fruiting Vegetables

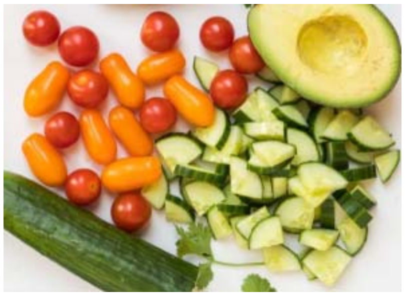

Which of the FRESH VEGETABLES listed below did you eat during the three months prior to conception? Check all that apply.

- ☑ Tomatoes, larger varieties (e.g. Beefsteak, Jersey & Big Boy)
- ☑ Tomatoes, bite-sized (e.g. Cherry & Grape)
- ☑ Avocado
- ☑ Cucumber
- ☑ Corn
- ☑ Sweet Bell Peppers (green, red or yellow)
- ☑ Hot Peppers
- ☑ Eggplant
- ☑ Summer Squash (e.g. zucchini & yellow squash)
- ☑ Winter Squash (e.g. acorn, butternut & spaghetti squash)
- ☐ None

Fresh Tomatoes, Large Variety

Serving Size of Tomato Large Variety: 1 tomato about 3" diameter

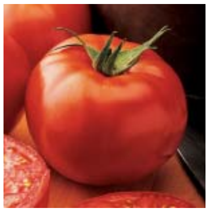

During the three months prior to conception, on average, how many servings of fresh tomatoes (large variety) did you consume per week.

- ○ 1 or less servings per week
- ○ 2 to 4 servings per week
- ○ 5 to 7 servings per week
- ○ 8 to 10 servings per week
- ○ 11 to 13 servings per week
- ○ 14 or more servings per week

reset

Please indicate what percentage of the fresh tomatoes (large variety) you consumed were organic.

- ○ 0% to 10%
- ○ 11% to 33%
- ○ 33% to 66%
- ○ 67% to 90%
- ○ 90% to 100%

reset

Protocol 1906366316 IRB Approved

Fresh Tomatoes, Bite-Sized

Serving Size of Tomato Bite-Sized: 1 cup or 27 tomatoes

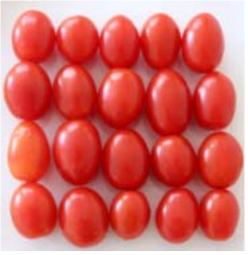

During the three months prior to conception, on average,
how many servings of fresh tomatoes (bite-sized) did you consume per week.

- 1 or less servings per week
- 2 to 4 servings per week
- 5 to 7 servings per week
- 8 to 10 servings per week
- 11 to 13 servings per week
- 14 or more servings per week

reset

Please indicate what percentage of the fresh tomatoes (bite-sized) you consumed were organic.

- 0% to 10%
- 11% to 33%
- 33% to 66%
- 67% to 90%
- 90% to 100%

reset

Fresh Avocado

Serving Size of Avocado: 1/3 medium avocado

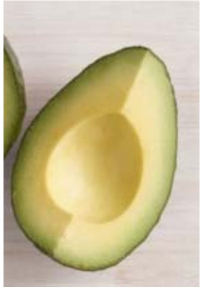

During the three months prior to conception, on average,
how many servings of fresh avocado did you consume per week.

- 1 or less servings per week
- 2 to 4 servings per week
- 5 to 7 servings per week
- 8 to 10 servings per week
- 11 to 13 servings per week
- 14 or more servings per week

reset

Protocol 1906366316 IRB Approved

Please indicate what percentage of the fresh avocado you consumed were organic.

- 0% to 10%
- 11% to 33%
- 33% to 66%
- 67% to 90%
- 90% to 100%

reset

Fresh Cucumber

Serving Size of Cucumber: 1 cup sliced or 1/2 small cucumber

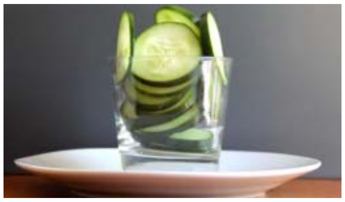

**During the three months prior to conception, on average,
how many servings of fresh cucumber did you consume per week.**

- 1 or less servings per week
- 2 to 4 servings per week
- 5 to 7 servings per week
- 8 to 10 servings per week
- 11 to 13 servings per week
- 14 or more servings per week

reset

Please indicate what percentage of the fresh cucumber you consumed were organic.

- 0% to 10%
- 11% to 33%
- 33% to 66%
- 67% to 90%
- 90% to 100%

reset

Fresh Corn

Serving Size of Corn: 1 large ear (8" - 9" long)

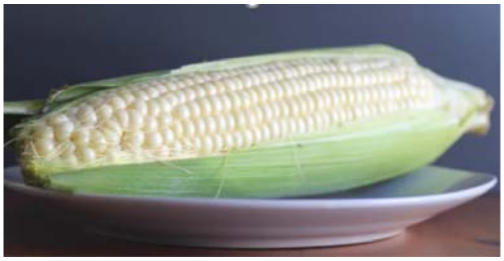

Protocol 1906366316 IRB Approved

During the three months prior to conception, on average, how many servings of fresh corn did you consume per week.

- 1 or less servings per week
- 2 to 4 servings per week
- 5 to 7 servings per week
- 8 to 10 servings per week
- 11 to 13 servings per week
- 14 or more servings per week

reset

Please indicate what percentage of the fresh corn you consumed were organic.

- 0% to 10%
- 11% to 33%
- 33% to 66%
- 67% to 90%
- 90% to 100%

reset

Fresh Sweet Bell Peppers

Serving Size of Sweet Bell Peppers: 1 large pepper (3" diameter, 3¾" long) or 1 cup chopped

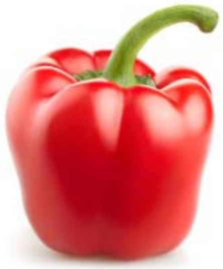

During the three months prior to conception, on average, how many servings of fresh sweet bell peppers did you consume per week.

- 1 or less servings per week
- 2 to 4 servings per week
- 5 to 7 servings per week
- 8 to 10 servings per week
- 11 to 13 servings per week
- 14 or more servings per week

reset

Please indicate what percentage of the fresh sweet bell peppers you consumed were organic.

- 0% to 10%
- 11% to 33%
- 33% to 66%
- 67% to 90%
- 90% to 100%

reset

Protocol 1906366316 IRB Approved

Fresh Hot Peppers

Serving Size of Hot Peppers: 1 medium hot pepper

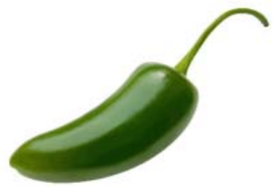

**During the three months prior to conception, on average,
how many servings of fresh hot peppers did you consume per week.**

- 1 or less servings per week
- 2 to 4 servings per week
- 5 to 7 servings per week
- 8 to 10 servings per week
- 11 to 13 servings per week
- 14 or more servings per week

reset

Please indicate what percentage of the fresh hot peppers you consumed were organic.

- 0% to 10%
- 11% to 33%
- 33% to 66%
- 67% to 90%
- 90% to 100%

reset

Fresh Eggplant

Serving Size: 1/5 large eggplant

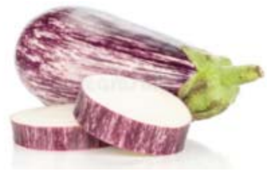

**During the three months prior to conception, on average,
how many servings of fresh eggplant did you consume per week.**

- 1 or less servings per week
- 2 to 4 servings per week
- 5 to 7 servings per week
- 8 to 10 servings per week
- 11 to 13 servings per week
- 14 or more servings per week

reset

Protocol 1906366316 IRB Approved

Please indicate what percentage of the fresh eggplant you consumed were organic.

- ◯ 0% to 10%
- ◯ 11% to 33%
- ◯ 33% to 66%
- ◯ 67% to 90%
- ◯ 90% to 100%

reset

Fresh Summer Squash (ex. zucchini & yellow squash)

Serving Size of Summer Squash: (1 cup chopped or 1/2 medium squash)

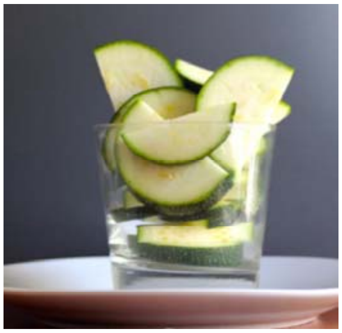

During the three months prior to conception, on average, how many servings of fresh summer squash did you consume per week.

- ◯ 1 or less servings per week
- ◯ 2 to 4 servings per week
- ◯ 5 to 7 servings per week
- ◯ 8 to 10 servings per week
- ◯ 11 to 13 servings per week
- ◯ 14 or more servings per week

reset

Please indicate what percentage of the fresh summer squash you consumed were organic.

- ◯ 0% to 10%
- ◯ 11% to 33%
- ◯ 33% to 66%
- ◯ 67% to 90%
- ◯ 90% to 100%

reset

Fresh Winter Squash (ex. acorn, butternut & spaghetti squash)

Serving Size: 1/2 acorn squash or 1 cup cubed winter squash

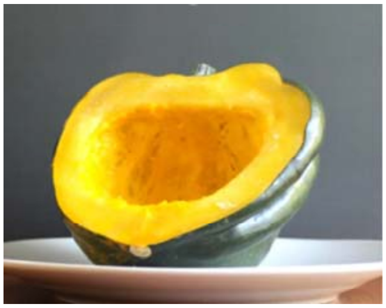

Protocol 1906366316 IRB Approved

During the three months prior to conception, on average, how many servings of fresh winter squash did you consume per week.

- ◯ 1 or less servings per week
- ◯ 2 to 4 servings per week
- ◯ 5 to 7 servings per week
- ◯ 8 to 10 servings per week
- ◯ 11 to 13 servings per week
- ◯ 14 or more servings per week

reset

Please indicate what percentage of the fresh winter squash you consumed were organic.

- ◯ 0% to 10%
- ◯ 11% to 33%
- ◯ 33% to 66%
- ◯ 67% to 90%
- ◯ 90% to 100%

reset

FRESH VEGETABLES - Peas & Root Vegetables

Peas & Root Vegetables

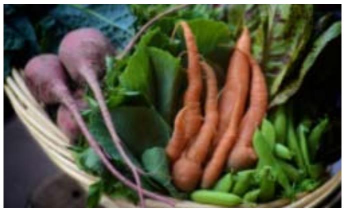

Which of the FRESH VEGETABLES listed below did you eat during the three months prior to conception? Check all that apply.

- ☑ Green Beans (Raw or Cooked)
- ☑ Snap Peas (Raw or Cooked)
- ☑ Sweet Peas (Raw or Cooked)
- ☑ Carrots (Raw or Cooked)
- ☑ Potatoes (Baked, Boiled or Mashed)
- ☑ Sweet Potatoes (Baked, Boiled or Mashed)
- ☐ None

Fresh Green Beans

Serving Size: 1 cup cooked

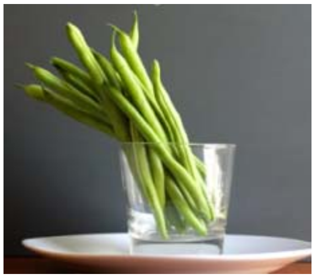

Protocol 1906366316 IRB Approved

During the three months prior to conception, on average,
how many servings of fresh green beans did you consume per week.

- 1 or less servings per week
- 2 to 4 servings per week
- 5 to 7 servings per week
- 8 to 10 servings per week
- 11 to 13 servings per week
- 14 or more servings per week

reset

Please indicate what percentage of the fresh green beans you consumed were organic.

- 0% to 10%
- 11% to 33%
- 33% to 66%
- 67% to 90%
- 90% to 100%

reset

Fresh Snap Peas

Serving Size: 1 cup

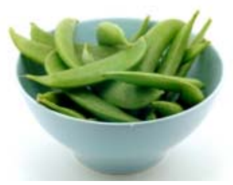

During the three months prior to conception, on average,
how many servings of fresh snap peas did you consume per week.

- 1 or less servings per week
- 2 to 4 servings per week
- 5 to 7 servings per week
- 8 to 10 servings per week
- 11 to 13 servings per week
- 14 or more servings per week

reset

Please indicate what percentage of the fresh snap peas you consumed were organic.

- 0% to 10%
- 11% to 33%
- 33% to 66%
- 67% to 90%
- 90% to 100%

reset

Protocol 1906366316 IRB Approved

Fresh Sweet Peas

Serving Size: 1 cup

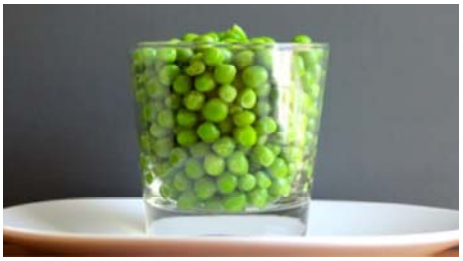

During the three months prior to conception, on average, how many servings of fresh sweet peas did you consume per week.

- ◯ 1 or less servings per week
- ◯ 2 to 4 servings per week
- ◯ 5 to 7 servings per week
- ◯ 8 to 10 servings per week
- ◯ 11 to 13 servings per week
- ◯ 14 or more servings per week

reset

Please indicate what percentage of the fresh sweet peas you consumed were organic.

- ◯ 0% to 10%
- ◯ 11% to 33%
- ◯ 33% to 66%
- ◯ 67% to 90%
- ◯ 90% to 100%

reset

Fresh Carrots

Serving Size: 1 cup chopped or 2 medium carrots or 12 -15 baby carrots

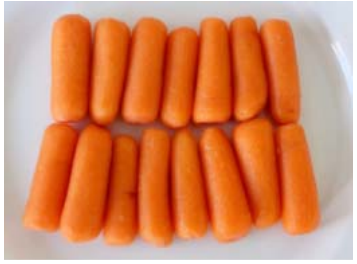

During the three months prior to conception, on average, how many servings of fresh carrots did you consume per week.

- ◯ 1 or less servings per week
- ◯ 2 to 4 servings per week
- ◯ 5 to 7 servings per week
- ◯ 8 to 10 servings per week
- ◯ 11 to 13 servings per week
- ◯ 14 or more servings per week

reset

Protocol 1906366316 IRB Approved

Please indicate what percentage of the fresh carrots you consumed were organic.

- 0% to 10%
- 11% to 33%
- 33% to 66%
- 67% to 90%
- 90% to 100%

reset

Fresh Potatoes

Serving Size: 1 medium potato or 1 cup diced

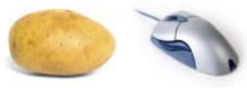

During the three months prior to conception, on average,
how many servings of fresh potatoes did you consume per week.

- 1 or less servings per week
- 2 to 4 servings per week
- 5 to 7 servings per week
- 8 to 10 servings per week
- 11 to 13 servings per week
- 14 or more servings per week

reset

Please indicate what percentage of the fresh potatoes you consumed were organic.

- 0% to 10%
- 11% to 33%
- 33% to 66%
- 67% to 90%
- 90% to 100%

reset

Fresh Sweet Potatoes

Serving Size: 1 large sweet potato or 1 cup mashed or diced

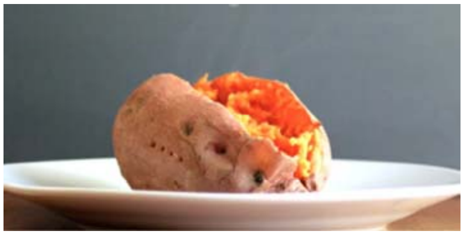

Protocol 1906366316 IRB Approved

**During the three months prior to conception, on average,
how many servings of fresh sweet potatoes did you consume per week.**

- ◯ 1 or less servings per week
- ◯ 2 to 4 servings per week
- ◯ 5 to 7 servings per week
- ◯ 8 to 10 servings per week
- ◯ 11 to 13 servings per week
- ◯ 14 or more servings per week

reset

Please indicate what percentage of the fresh sweet potatoes you consumed were organic.

- ◯ 0% to 10%
- ◯ 11% to 33%
- ◯ 33% to 66%
- ◯ 67% to 90%
- ◯ 90% to 100%

reset

BEANS - cooked dried or canned

Beans

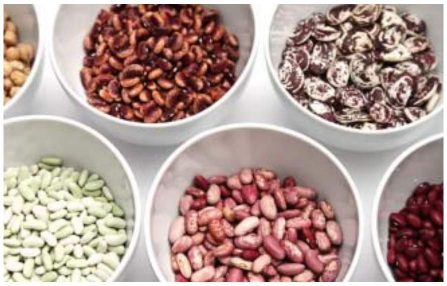

**Which of the BEANS (cooked dried or canned) listed below did you eat
during the three months prior to conception? Check all that apply.**

- ☑ Black Beans
- ☑ Garbanzo Beans
- ☑ Kidney Beans
- ☑ Pinto Beans
- ☐ None

Black Beans

Serving Size: 1 cup cooked

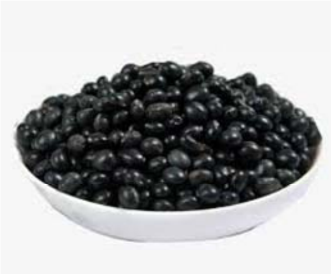

Protocol 1906366316 IRB Approved

During the three months prior to conception, on average, how many servings of black beans did you consume per week.

- 1 or less servings per week
- 2 to 4 servings per week
- 5 to 7 servings per week
- 8 to 10 servings per week
- 11 to 13 servings per week
- 14 or more servings per week

reset

Please indicate what percentage of the black beans you consumed were organic.

- 0% to 10%
- 11% to 33%
- 33% to 66%
- 67% to 90%
- 90% to 100%

reset

Garbanzo Beans

Serving Size: 1 cup cooked

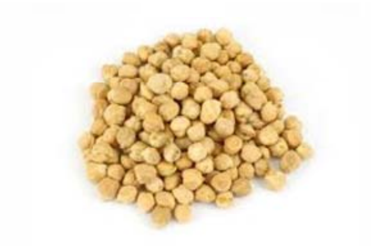

During the three months prior to conception, on average, how many servings of garbanzo beans did you consume per week.

- 1 or less servings per week
- 2 to 4 servings per week
- 5 to 7 servings per week
- 8 to 10 servings per week
- 11 to 13 servings per week
- 14 or more servings per week

reset

Please indicate what percentage of the garbanzo beans you consumed were organic.

- 0% to 10%
- 11% to 33%
- 33% to 66%
- 67% to 90%
- 90% to 100%

reset

Protocol 1906366316 IRB Approved

Kidney Beans

Serving Size: 1 cup cooked

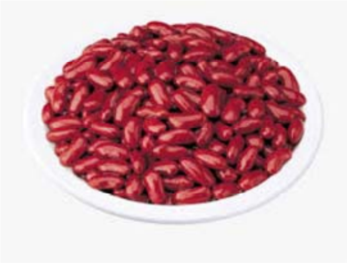

During the three months prior to conception, on average, how many servings of kidney beans did you consume per week.

- 1 or less servings per week
- 2 to 4 servings per week
- 5 to 7 servings per week
- 8 to 10 servings per week
- 11 to 13 servings per week
- 14 or more servings per week

reset

Please indicate what percentage of the kidney beans you consumed were organic.

- 0% to 10%
- 11% to 33%
- 33% to 66%
- 67% to 90%
- 90% to 100%

reset

Pinto Beans

Serving Size: 1 cup

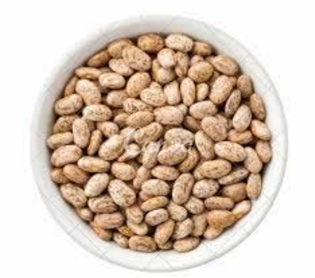

During the three months prior to conception, on average, how many servings of pinto beans did you consume per week.

- 1 or less servings per week
- 2 to 4 servings per week
- 5 to 7 servings per week
- 8 to 10 servings per week
- 11 to 13 servings per week
- 14 or more servings per week

reset

Protocol 1906366316 IRB Approved

Please indicate what percentage of the pinto beans you consumed were organic.

- ◯ 0% to 10%
- ◯ 11% to 33%
- ◯ 33% to 66%
- ◯ 67% to 90%
- ◯ 90% to 100%

reset

WHOLE GRAINS

Whole Grains

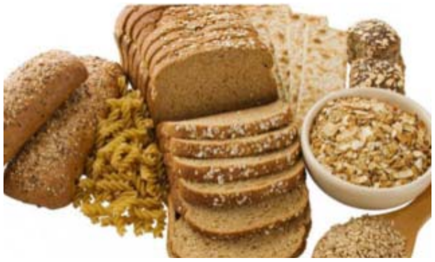

Which of the following foods listed below did you eat during the three months prior to conception? Check all that apply.

- ☑ Whole Wheat Breads (e.g. sandwich bread, muffins, english muffins, bagels, pancakes)
- ☑ Whole Grain Cereals (e.g. Fiber One, Shredded Wheat, Cheerios, Kashi, Wheaties, etc.)
- ☑ Oatmeal
- ☑ Granola Products (e.g. Granola, Granola Bars)
- ☑ Whole Grain Pasta
- ☑ Rice (ex. white rice, brown rice, wild rice)
- ☑ Corn Snacks (e.g. Popcorn, Tortilla Chips or Corn Chips)
- ☑ Barley
- ☑ Soy Products (e.g. Tofu, Tempeh, Soy Nuts, Soy Grains, etc.)
- ☐ None

Whole Wheat Breads (slice, muffin, waffle, tortilla, english muffin)

Serving Size of Whole Wheat Bread: 1 regular slice of bread or 1 item (ex. small muffin, waffle, tortilla, english muffin)

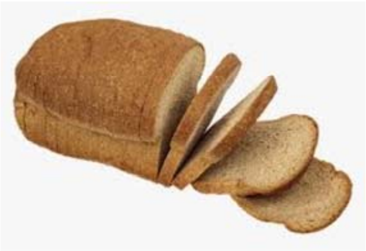

Protocol 1906366316 IRB Approved

**During the three months prior to conception, on average,
how many servings of whole wheat breads did you consume per week.**

- 1 or less servings per week
- 2 to 4 servings per week
- 5 to 7 servings per week
- 8 to 10 servings per week
- 11 to 13 servings per week
- 14 or more servings per week

reset

Please indicate what percentage of the whole wheat breads you consumed were organic.

- 0% to 10%
- 11% to 33%
- 33% to 66%
- 67% to 90%
- 90% to 100%

reset

Whole Grain Cereals

Serving Size of Whole Grain Cereals: 1 cup or 1 medium bowl of cereal

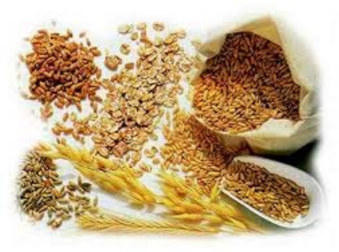

**During the three months prior to conception, on average,
how many servings of whole grain cereals did you consume per week.**

- 1 or less servings per week
- 2 to 4 servings per week
- 5 to 7 servings per week
- 8 to 10 servings per week
- 11 to 13 servings per week
- 14 or more servings per week

reset

Please indicate what percentage of the whole wheat breads you consumed were organic.

- 0% to 10%
- 11% to 33%
- 33% to 66%
- 67% to 90%
- 90% to 100%

reset

Protocol 1906366316 IRB Approved

Oatmeal

Serving Size of Oatmeal: 1/2 cup cooked, 1 packet instant, or 1/3 cup dry

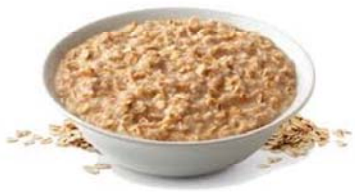

During the three months prior to conception, on average, how many servings of oatmeal did you consume per week.

- ◯ 1 or less servings per week
- ◯ 2 to 4 servings per week
- ◯ 5 to 7 servings per week
- ◯ 8 to 10 servings per week
- ◯ 11 to 13 servings per week
- ◯ 14 or more servings per week

reset

Please indicate what percentage of the oatmeal you consumed were organic.

- ◯ 0% to 10%
- ◯ 11% to 33%
- ◯ 33% to 66%
- ◯ 67% to 90%
- ◯ 90% to 100%

reset

Granola Products (ex. Granola, Granola Bars)

Serving Size of Granola Bars: 1 cup or 1 bar

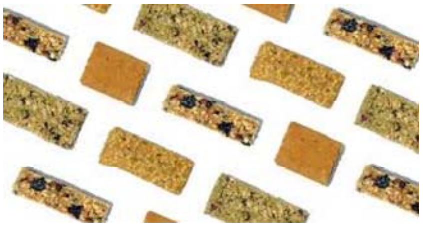

During the three months prior to conception, on average, how many servings of granola did you consume per week.

- ◯ 1 or less servings per week
- ◯ 2 to 4 servings per week
- ◯ 5 to 7 servings per week
- ◯ 8 to 10 servings per week
- ◯ 11 to 13 servings per week
- ◯ 14 or more servings per week

reset

Protocol 1906366316 IRB Approved

Please indicate what percentage of the granola products you consumed were organic.

- 0% to 10%
- 11% to 33%
- 33% to 66%
- 67% to 90%
- 90% to 100%

reset

Whole Grain Pasta

Serving Size of Whole Grain Pasta: 1/2 cup cooked

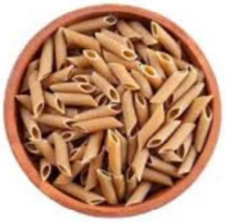

**During the three months prior to conception, on average,
how many servings of whole grain pasta did you consume per week.**

- 1 or less servings per week
- 2 to 4 servings per week
- 5 to 7 servings per week
- 8 to 10 servings per week
- 11 to 13 servings per week
- 14 or more servings per week

reset

Please indicate what percentage of the whole grain pasta you consumed were organic.

- 0% to 10%
- 11% to 33%
- 33% to 66%
- 67% to 90%
- 90% to 100%

reset

Rice

Serving Size of Rice: 1/2 cup cooked

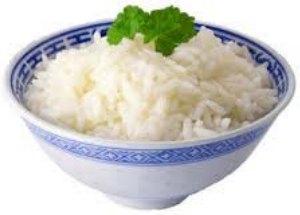

Protocol 1906366316 IRB Approved

During the three months prior to conception, on average, how many servings of rice did you consume per week.

- 1 or less servings per week
- 2 to 4 servings per week
- 5 to 7 servings per week
- 8 to 10 servings per week
- 11 to 13 servings per week
- 14 or more servings per week

reset

Please indicate what percentage of the rice you consumed were organic.

- 0% to 10%
- 11% to 33%
- 33% to 66%
- 67% to 90%
- 90% to 100%

reset

Corn-based Snacks (ex. Popcorn, Tortilla Chips or Corn Chips)

Serving Size: 1 cup

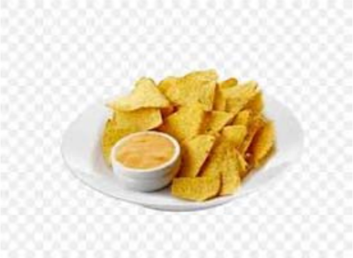

During the three months prior to conception, on average, how many servings of corn-based snacks did you consume per week.

- 1 or less servings per week
- 2 to 4 servings per week
- 5 to 7 servings per week
- 8 to 10 servings per week
- 11 to 13 servings per week
- 14 or more servings per week

reset

Please indicate what percentage of the corn-based snacks you consumed were organic.

- 0% to 10%
- 11% to 33%
- 33% to 66%
- 67% to 90%
- 90% to 100%

reset

Protocol 1906366316 IRB Approved

Barley

Serving Size: 1 cup cooked

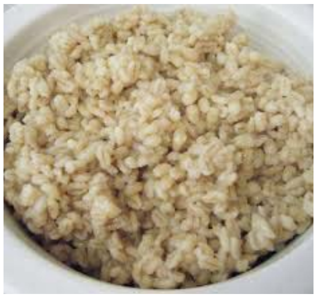

During the three months prior to conception, on average, how many servings of barley did you consume per week.

- 1 or less servings per week
- 2 to 4 servings per week
- 5 to 7 servings per week
- 8 to 10 servings per week
- 11 to 13 servings per week
- 14 or more servings per week

reset

Please indicate what percentage of barley you consumed was organic.

- 0% to 10%
- 11% to 33%
- 33% to 66%
- 67% to 90%
- 90% to 100%

reset

Soy Products (e.g. Tufu, Tempeh, Soy Grain, etc.)

Serving Size: 1/4 cup

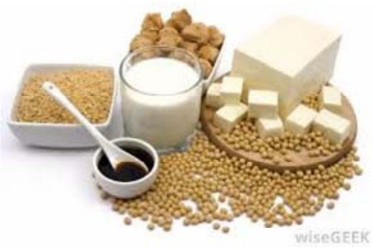

During the three months prior to conception, on average, how many servings of soy products did you consume per week.

- 1 or less servings per week
- 2 to 4 servings per week
- 5 to 7 servings per week
- 8 to 10 servings per week
- 11 to 13 servings per week
- 14 or more servings per week

reset

Protocol 1906366316 IRB Approved

Please indicate what percentage of soy products you consumed was organic.

- 0% to 10%
- 11% to 33%
- 33% to 66%
- 67% to 90%
- 90% to 100%

reset

NUTS & OILS

Nuts & Oils

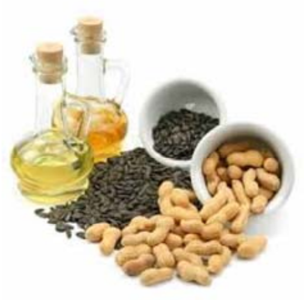

Which of the following nuts and oils listed below did you eat during the three months prior to conception? Check all that apply.

- [x] Almonds
- [x] Almond Butter
- [x] Peanut Butter
- [ ] None

Almonds

Serving Size: 12 whole almonds

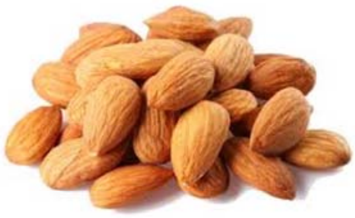

During the three months prior to conception, on average, how many servings of almonds did you consume per week.

- 1 or less servings per week
- 2 to 4 servings per week
- 5 to 7 servings per week
- 8 to 10 servings per week
- 11 to 13 servings per week
- 14 or more servings per week

reset

Protocol 1906366316 IRB Approved

Please indicate what percentage of almonds you consumed was organic.

- 0% to 10%
- 11% to 33%
- 33% to 66%
- 67% to 90%
- 90% to 100%

reset

Almond Butter

Serving Size: 1 tablespoon

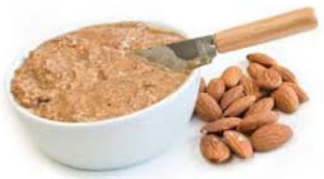

During the three months prior to conception, on average, how many servings of almond butter did you consume per week.

- 1 or less servings per week
- 2 to 4 servings per week
- 5 to 7 servings per week
- 8 to 10 servings per week
- 11 to 13 servings per week
- 14 or more servings per week

reset

Please indicate what percentage of almond butter you consumed was organic.

- 0% to 10%
- 11% to 33%
- 33% to 66%
- 67% to 90%
- 90% to 100%

reset

Peanut Butter

Serving Size: 1 tablespoon

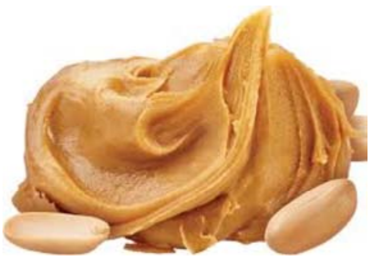

Protocol 1906366316 IRB Approved

During the three months prior to conception, on average, how many servings of peanut butter did you consume per week.

- ○ 1 or less servings per week
- ○ 2 to 4 servings per week
- ○ 5 to 7 servings per week
- ○ 8 to 10 servings per week
- ○ 11 to 13 servings per week
- ○ 14 or more servings per week

reset

Please indicate what percentage of peanut butter you consumed was organic.

- ○ 0% to 10%
- ○ 11% to 33%
- ○ 33% to 66%
- ○ 67% to 90%
- ○ 90% to 100%

reset

DRIED/DEHYDRATED FRUITS

Dried Dehydrated Fruits

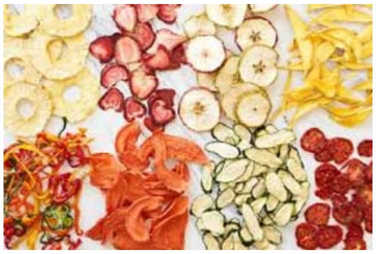

Which of the following dried/dehydrated fruits listed below did you eat during the three months prior to conception? Check all that apply.

- ☑ Raisins
- ☑ Dried/Dehydrated Apples
- ☑ Dried/Dehydrated Plums (Prunes)
- ☑ Dried/Dehydrated Mangoes
- ☑ Dried/Dehydrated Cranberries
- ☑ Dried/Dehydrated Apricots
- ☑ Dried/Dehydrated Dates
- ☐ None

Protocol 1906366316 IRB Approved

Raisins

Serving Size: 1 small box (1.5 oz)

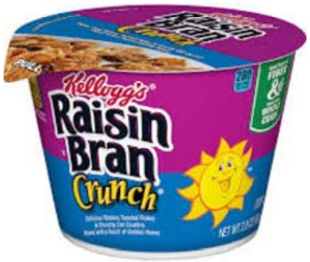

During the three months prior to conception, on average, how many servings of raisins did you consume per week.

- ◯ 1 or less servings per week
- ◯ 2 to 4 servings per week
- ◯ 5 to 7 servings per week
- ◯ 8 to 10 servings per week
- ◯ 11 to 13 servings per week
- ◯ 14 or more servings per week

reset

Please indicate what percentage of raisins you consumed was organic.

- ◯ 0% to 10%
- ◯ 11% to 33%
- ◯ 33% to 66%
- ◯ 67% to 90%
- ◯ 90% to 100%

reset

Dried/Dehydrated Apples

Serving Size: 1/2 cup

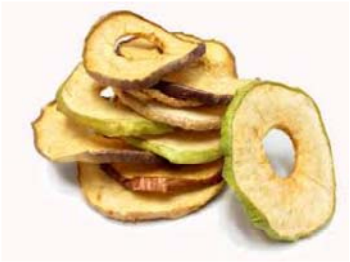

During the three months prior to conception, on average, how many servings of dried/dehydrated apples did you consume per week.

- ◯ 1 or less servings per week
- ◯ 2 to 4 servings per week
- ◯ 5 to 7 servings per week
- ◯ 8 to 10 servings per week
- ◯ 11 to 13 servings per week
- ◯ 14 or more servings per week

reset

Protocol 1906366316 IRB Approved

Please indicate what percentage of dried/dehydrated apples you consumed was organic.

- 0% to 10%
- 11% to 33%
- 33% to 66%
- 67% to 90%
- 90% to 100%

reset

Dried/Dehydrated Plums (Prunes)

Serving Size: 1/2 cup

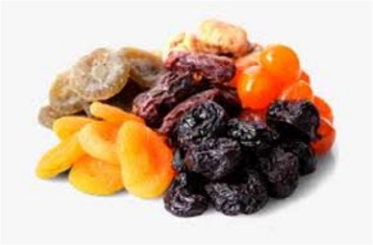

**During the three months prior to conception, on average,
how many servings of dried/dehydrated plums did you consume per week.**

- 1 or less servings per week
- 2 to 4 servings per week
- 5 to 7 servings per week
- 8 to 10 servings per week
- 11 to 13 servings per week
- 14 or more servings per week

reset

Please indicate what percentage of dried/dehydrated plums you consumed was organic.

- 0% to 10%
- 11% to 33%
- 33% to 66%
- 67% to 90%
- 90% to 100%

reset

Dried/Dehydrated Mangoes

Serving Size: 1/2 cup

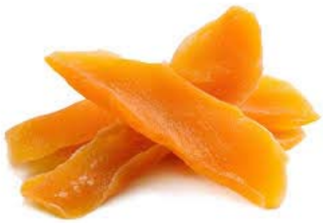

Protocol 1906366316 IRB Approved

During the three months prior to conception, on average, how many servings of dried/dehydrated mangoes did you consume per week.

- 1 or less servings per week
- 2 to 4 servings per week
- 5 to 7 servings per week
- 8 to 10 servings per week
- 11 to 13 servings per week
- 14 or more servings per week

reset

Please indicate what percentage of dried/dehydrated mangoes you consumed was organic.

- 0% to 10%
- 11% to 33%
- 33% to 66%
- 67% to 90%
- 90% to 100%

reset

Dried/Dehydrated Cranberries

Serving Size: 1/2 cup

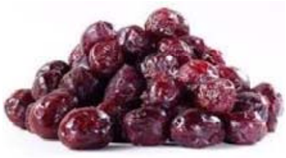

During the three months prior to conception, on average, how many servings of dried/dehydrated cranberries did you consume per week.

- 1 or less servings per week
- 2 to 4 servings per week
- 5 to 7 servings per week
- 8 to 10 servings per week
- 11 to 13 servings per week
- 14 or more servings per week

reset

Please indicate what percentage of dried/dehydrated cranberries you consumed was organic.

- 0% to 10%
- 11% to 33%
- 33% to 66%
- 67% to 90%
- 90% to 100%

reset

Protocol 1906366316 IRB Approved

Dried/Dehydrated Apricots

Serving Size: 1/2 cup

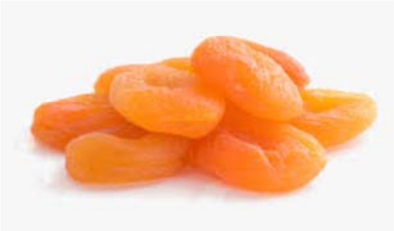

During the three months prior to conception, on average, how many servings of dried/dehydrated apricots did you consume per week.

- 1 or less servings per week
- 2 to 4 servings per week
- 5 to 7 servings per week
- 8 to 10 servings per week
- 11 to 13 servings per week
- 14 or more servings per week

reset

Please indicate what percentage of dried/dehydrated apricots you consumed was organic.

- 0% to 10%
- 11% to 33%
- 33% to 66%
- 67% to 90%
- 90% to 100%

reset

Dried/Dehydrated Dates

Serving Size: 1/2 cup

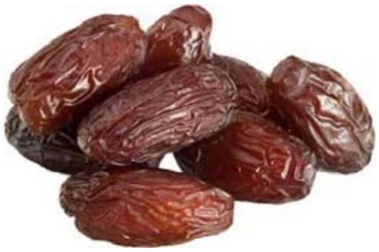

During the three months prior to conception, on average, how many servings of dried/dehydrated dates did you consume per week.

- 1 or less servings per week
- 2 to 4 servings per week
- 5 to 7 servings per week
- 8 to 10 servings per week
- 11 to 13 servings per week
- 14 or more servings per week

reset

Protocol 1906366316 IRB Approved

Please indicate what percentage of dried/dehydrated dates you consumed was organic.

- ◯ 0% to 10%
- ◯ 11% to 33%
- ◯ 33% to 66%
- ◯ 67% to 90%
- ◯ 90% to 100%

reset

JUICES

Juices

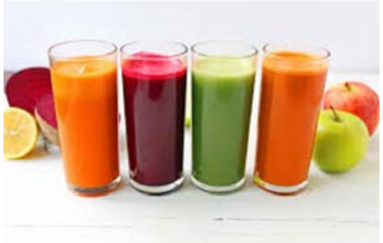

Which of the following fruit juces listed below did you drink during the three months prior to conception? Check all that apply.

- ☑ Apple Juice
- ☑ Orange Juice
- ☑ Cranberry Juice
- ☑ Grapefruit Juice
- ☑ Lemonade Juice
- ☑ Pineapple Juice
- ☑ Grape juice
- ☑ Mix of different fruits and/or vegetables juice
- ☐ None

Apple Juice

Serving Size: 8 fl oz (1 cup)

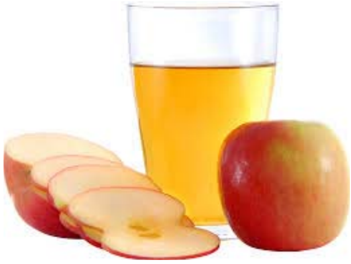

Protocol 1906366316 IRB Approved

**During the three months prior to conception, on average,
how many servings of apple juice did you consume per week.**

- 1 or less servings per week
- 2 to 4 servings per week
- 5 to 7 servings per week
- 8 to 10 servings per week
- 11 to 13 servings per week
- 14 or more servings per week

reset

Please indicate what percentage of apple juice you consumed was organic.

- 0% to 10%
- 11% to 33%
- 33% to 66%
- 67% to 90%
- 90% to 100%

reset

Orange Juice

Serving Size: 8 fl oz (1 cup)

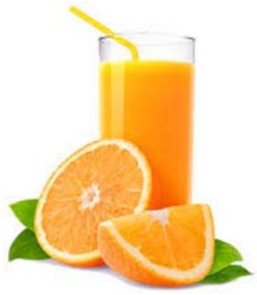

**During the three months prior to conception, on average,
how many servings of orange juice did you consume per week.**

- 1 or less servings per week
- 2 to 4 servings per week
- 5 to 7 servings per week
- 8 to 10 servings per week
- 11 to 13 servings per week
- 14 or more servings per week

reset

Please indicate what percentage of orange juice you consumed was organic.

- 0% to 10%
- 11% to 33%
- 33% to 66%
- 67% to 90%
- 90% to 100%

reset

Protocol 1906366316 IRB Approved

Cranberry Juice

Serving Size: 8 fl oz (1 cup)

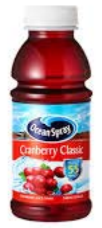

During the three months prior to conception, on average, how many servings of cranberry juice did you consume per week.

- 1 or less servings per week
- 2 to 4 servings per week
- 5 to 7 servings per week
- 8 to 10 servings per week
- 11 to 13 servings per week
- 14 or more servings per week

reset

Please indicate what percentage of cranberry juice you consumed was organic.

- 0% to 10%
- 11% to 33%
- 33% to 66%
- 67% to 90%
- 90% to 100%

reset

Grapefruit Juice

Serving Size: 8 fl oz (1 cup)

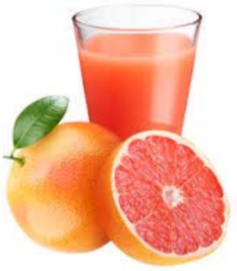

During the three months prior to conception, on average, how many servings of grapefruit juice did you consume per week.

- 1 or less servings per week
- 2 to 4 servings per week
- 5 to 7 servings per week
- 8 to 10 servings per week
- 11 to 13 servings per week
- 14 or more servings per week

reset

Protocol 1906366316 IRB Approved

Please indicate what percentage of grapefruit juice you consumed was organic.

- 0% to 10%
- 11% to 33%
- 33% to 66%
- 67% to 90%
- 90% to 100%

reset

Lemonade

Serving Size: 8 fl oz (1 cup)

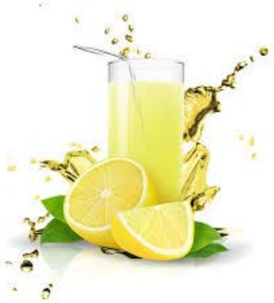

During the three months prior to conception, on average, how many servings of lemonade did you consume per week.

- 1 or less servings per week
- 2 to 4 servings per week
- 5 to 7 servings per week
- 8 to 10 servings per week
- 11 to 13 servings per week
- 14 or more servings per week

reset

Please indicate what percentage of lemonade you consumed was organic.

- 0% to 10%
- 11% to 33%
- 33% to 66%
- 67% to 90%
- 90% to 100%

reset

Pineapple Juice

Serving Size: 8 fl oz (1 cup)

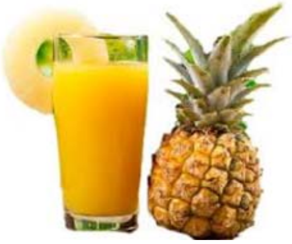

Protocol 1906366316 IRB Approved

During the three months prior to conception, on average, how many servings of pineapple juice did you consume per week.

- 1 or less servings per week
- 2 to 4 servings per week
- 5 to 7 servings per week
- 8 to 10 servings per week
- 11 to 13 servings per week
- 14 or more servings per week

reset

Please indicate what percentage of pineapple juice you consumed was organic.

- 0% to 10%
- 11% to 33%
- 33% to 66%
- 67% to 90%
- 90% to 100%

reset

Grape Juice

Serving Size: 8 fl oz (1 cup)

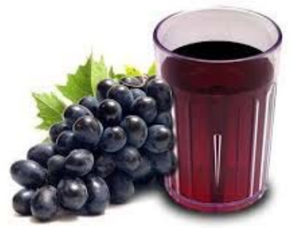

During the three months prior to conception, on average, how many servings of grape juice did you consume per week.

- 1 or less servings per week
- 2 to 4 servings per week
- 5 to 7 servings per week
- 8 to 10 servings per week
- 11 to 13 servings per week
- 14 or more servings per week

reset

Please indicate what percentage of grape juice you consumed was organic.

- 0% to 10%
- 11% to 33%
- 33% to 66%
- 67% to 90%
- 90% to 100%

reset

Protocol 1906366316 IRB Approved

Mix of different fruits and/or vegetable juice

Serving Size: 8 fl oz (1 cup)

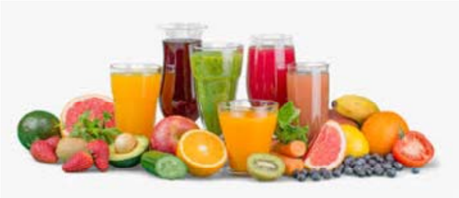

During the three months prior to conception, on average, how many servings of mixed juice did you consume per week.

- ◯ 1 or less servings per week
- ◯ 2 to 4 servings per week
- ◯ 5 to 7 servings per week
- ◯ 8 to 10 servings per week
- ◯ 11 to 13 servings per week
- ◯ 14 or more servings per week

reset

Please indicate what percentage of mixed juice you consumed was organic.

- ◯ 0% to 10%
- ◯ 11% to 33%
- ◯ 33% to 66%
- ◯ 67% to 90%
- ◯ 90% to 100%

reset

SAUCES & SOUPS

Souces and Soups

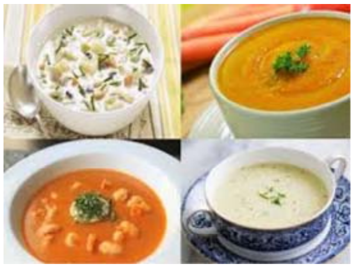

Which of the following dried/dehydrated fruits listed below did you eat during the three months prior to conception? Check all that apply.

- ☑ Apple Sauce / Puree
- ☑ Pear Puree
- ☑ Tomato Sauce
- ☑ Canned Tomato - Whole or Crushed
- ☑ Tomato Soup
- ☐ None

Protocol 1906366316 IRB Approved

Apple Sauce and/or Apple Puree

Serving Size: 1 cup

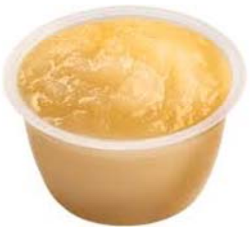

During the three months prior to conception, on average, how many servings of apple sauce &/or apple puree did you consume per week.

- 1 or less servings per week
- 2 to 4 servings per week
- 5 to 7 servings per week
- 8 to 10 servings per week
- 11 to 13 servings per week
- 14 or more servings per week

reset

Please indicate what percentage of apple sauce &/or apple puree you consumed was organic.

- 0% to 10%
- 11% to 33%
- 33% to 66%
- 67% to 90%
- 90% to 100%

reset

Pear Puree

Serving Size: 1 cup

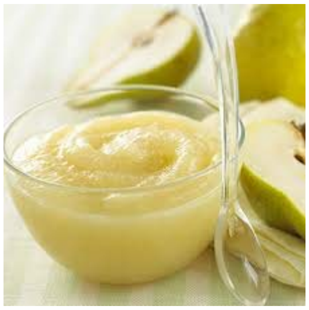

During the three months prior to conception, on average, how many servings of pear puree did you consume per week.

- 1 or less servings per week
- 2 to 4 servings per week
- 5 to 7 servings per week
- 8 to 10 servings per week
- 11 to 13 servings per week
- 14 or more servings per week

reset

Protocol 1906366316 IRB Approved

Please indicate what percentage of pear puree you consumed was organic.

- 0% to 10%
- 11% to 33%
- 33% to 66%
- 67% to 90%
- 90% to 100%

reset

Tomato Sauce

Serving Size: 1 cup

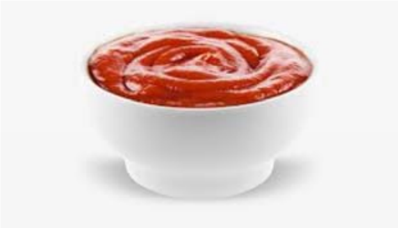

During the three months prior to conception, on average, how many servings of tomato sauce did you consume per week.

- 1 or less servings per week
- 2 to 4 servings per week
- 5 to 7 servings per week
- 8 to 10 servings per week
- 11 to 13 servings per week
- 14 or more servings per week

reset

Please indicate what percentage of tomato sauce you consumed was organic.

- 0% to 10%
- 11% to 33%
- 33% to 66%
- 67% to 90%
- 90% to 100%

reset

Canned Tomatoes -Crushed or Whole

Serving Size: 1 cup

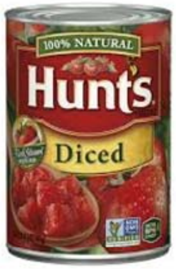

Protocol 1906366316 IRB Approved

**During the three months prior to conception, on average,
how many servings of canned tomatoes - crushed or whole did you consume per week.**

- 1 or less servings per week
- 2 to 4 servings per week
- 5 to 7 servings per week
- 8 to 10 servings per week
- 11 to 13 servings per week
- 14 or more servings per week

reset

Please indicate what percentage of canned tomatoes - crushed or whole you consumed was organic.

- 0% to 10%
- 11% to 33%
- 33% to 66%
- 67% to 90%
- 90% to 100%

reset

Tomato Soup

Serving Size: 1 cup

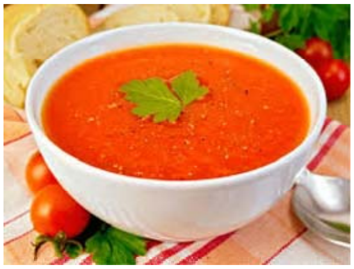

**During the three months prior to conception, on average,
how many servings of tomato soup did you consume per week.**

- 1 or less servings per week
- 2 to 4 servings per week
- 5 to 7 servings per week
- 8 to 10 servings per week
- 11 to 13 servings per week
- 14 or more servings per week

reset

Please indicate what percentage of tomato soup you consumed was organic.

- 0% to 10%
- 11% to 33%
- 33% to 66%
- 67% to 90%
- 90% to 100%

reset

FROZEN PRODUCTS

Protocol 1906366316 IRB Approved

Frozen Products

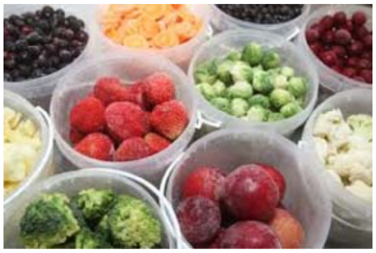

Which of the following frozen products listed below did you eat during the three months prior to conception? Check all that apply.

- ☑ Frozen or canned cherries
- ☑ Frozen or canned green beans
- ☑ Frozen or canned spinach
- ☐ None

Frozen Cherries

Serving Size: 1 cup

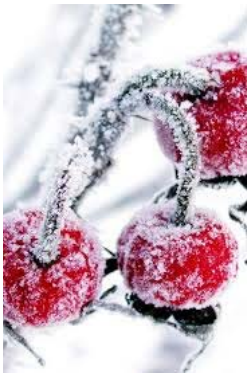

During the three months prior to conception, on average, how many servings of frozen cherries did you consume per week.

- ○ 1 or less servings per week
- ○ 2 to 4 servings per week
- ○ 5 to 7 servings per week
- ○ 8 to 10 servings per week
- ○ 11 to 13 servings per week
- ○ 14 or more servings per week

reset

Please indicate what percentage of frozen cherries you consumed was organic.

- ○ 0% to 10%
- ○ 11% to 33%
- ○ 33% to 66%
- ○ 67% to 90%
- ○ 90% to 100%

reset

Protocol 1906366316 IRB Approved

Frozen Green Beans

Serving Size: 1 cup

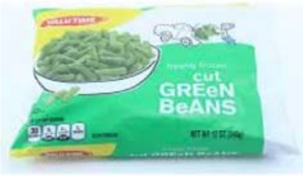

**During the three months prior to conception, on average,
how many servings of frozen green beans did you consume per week.**

- 1 or less servings per week
- 2 to 4 servings per week
- 5 to 7 servings per week
- 8 to 10 servings per week
- 11 to 13 servings per week
- 14 or more servings per week

reset

Please indicate what percentage of frozen green beans you consumed was organic.

- 0% to 10%
- 11% to 33%
- 33% to 66%
- 67% to 90%
- 90% to 100%

reset

Frozen Spinach

Serving Size: 1 cup

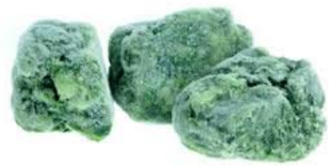

**During the three months prior to conception, on average,
how many servings of frozen spinach did you consume per week.**

- 1 or less servings per week
- 2 to 4 servings per week
- 5 to 7 servings per week
- 8 to 10 servings per week
- 11 to 13 servings per week
- 14 or more servings per week

reset

Protocol 1906366316 IRB Approved

Please indicate what percentage of frozen spinach you consumed was organic.

- ◯ 0% to 10%
- ◯ 11% to 33%
- ◯ 33% to 66%
- ◯ 67% to 90%
- ◯ 90% to 100%

reset

ANIMAL PRODUCTS

Animal Products

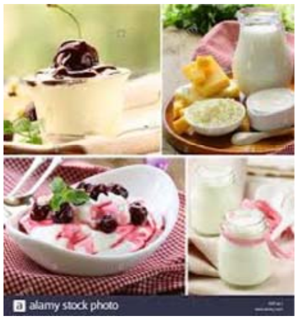

Which of the following animal products listed below did you consume during the three months prior to conception? Check all that apply.

- ☑ Milk (Dairy)
- ☑ Cheese (Dairy)
- ☑ Yogurt (Dairy)
- ☑ Ice Cream (Dairy)
- ☐ None

Milk (Dairy)

Serving Size: 1 cup

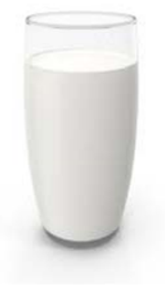

Protocol 1906366316 IRB Approved

During the three months prior to conception, on average, how many servings of milk did you consume per week.

- ◯ 1 or less servings per week
- ◯ 2 to 4 servings per week
- ◯ 5 to 7 servings per week
- ◯ 8 to 10 servings per week
- ◯ 11 to 13 servings per week
- ◯ 14 or more servings per week

reset

Please indicate what percentage of milk you consumed was organic.

- ◯ 0% to 10%
- ◯ 11% to 33%
- ◯ 33% to 66%
- ◯ 67% to 90%
- ◯ 90% to 100%

reset

Cheese (Dairy)

Serving Size: 1 slice American or 1/3 cup shredded

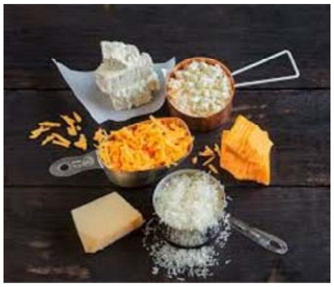

During the three months prior to conception, on average, how many servings of cheese did you consume per week.

- ◯ 1 or less servings per week
- ◯ 2 to 4 servings per week
- ◯ 5 to 7 servings per week
- ◯ 8 to 10 servings per week
- ◯ 11 to 13 servings per week
- ◯ 14 or more servings per week

reset

Please indicate what percentage of cheese you consumed was organic.

- ◯ 0% to 10%
- ◯ 11% to 33%
- ◯ 33% to 66%
- ◯ 67% to 90%
- ◯ 90% to 100%

reset

Protocol 1906366316 IRB Approved

Yogurt (Dairy)

Serving Size: 1 cup

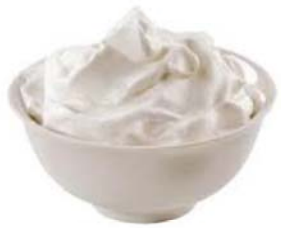

During the three months prior to conception, on average, how many servings of yogurt did you consume per week.

- ◯ 1 or less servings per week
- ◯ 2 to 4 servings per week
- ◯ 5 to 7 servings per week
- ◯ 8 to 10 servings per week
- ◯ 11 to 13 servings per week
- ◯ 14 or more servings per week

reset

Please indicate what percentage of yogurt you consumed was organic.

- ◯ 0% to 10%
- ◯ 11% to 33%
- ◯ 33% to 66%
- ◯ 67% to 90%
- ◯ 90% to 100%

reset

Ice Cream (Dairy)

Serving Size: 1 1/2 cups

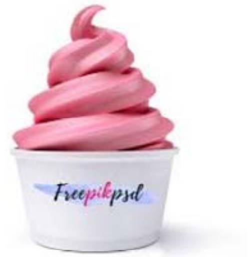

During the three months prior to conception, on average, how many servings of ice cream did you consume per week.

- ◯ 1 or less servings per week
- ◯ 2 to 4 servings per week
- ◯ 5 to 7 servings per week
- ◯ 8 to 10 servings per week
- ◯ 11 to 13 servings per week
- ◯ 14 or more servings per week

reset

Protocol 1906366316 IRB Approved

Please indicate what percentage of ice cream you consumed was organic.

- ◯ 0% to 10%
- ◯ 11% to 33%
- ◯ 33% to 66%
- ◯ 67% to 90%
- ◯ 90% to 100%

reset

Submit

Powered by REDCap

Protocol 1906366316 IRB Approved
